# Interactions among Norway spruce, the bark beetle *Ips typographus* and its fungal symbionts in times of drought

**DOI:** 10.1007/s10340-021-01341-y

**Published:** 2021-02-22

**Authors:** Sigrid Netherer, Dineshkumar Kandasamy, Anna Jirosová, Blanka Kalinová, Martin Schebeck, Fredrik Schlyter

**Affiliations:** 1 Department of Forest and Soil Sciences, University of Natural Resources and Life Sciences, BOKU, Vienna, Austria; 2 Department of Biochemistry, Max Planck Institute for Chemical Ecology, Jena, Germany; 3 ETM Faculty of Forestry and Wood Sciences, Czech University of Life Sciences, CULS, Praha-Suchdol, Czech Republic; 4 Chemical Ecology Plant Protection Department, Swedish University of Agricultural Sciences, SLU, Alnarp, Sweden

**Keywords:** *Picea abies*, Spruce bark beetle, Ophiostomatoid fungi, Drought, Tree defence, Specialised metabolites

## Abstract

Resilience and functionality of European Norway spruce forests are increasingly threatened by mass outbreaks of the bark beetle *Ips typographus* promoted by heat, wind throw and drought. Here, we review current knowledge on Norway spruce and *I. typographus* interactions from the perspective of drought-stressed trees, host selection, colonisation behaviour of beetles, with multi-level effects of symbiotic ophiostomatoid fungi. By including chemo-ecological, molecular and behavioural perspectives, we provide a comprehensive picture on this complex, multitrophic system in the light of climate change. Trees invest carbon into specialised metabolism to produce defence compounds against biotic invaders; processes that are strongly affected by physiological stress such as drought. Spruce bark contains numerous terpenoid and phenolic substances, which are important for bark beetle aggregation and attack success. Abiotic stressors such as increased temperatures and drought affect composition, amounts and emission rates of volatile compounds. Thus, drought events may influence olfactory responses of *I. typographus*, and further the pheromone communication enabling mass attack. In addition, *I. typographus* is associated with numerous ophiostomatoid fungal symbionts with multiple effects on beetle life history. Symbiotic fungi degrade spruce toxins, help to exhaust tree defences, produce beetle semiochemicals, and possibly provide nutrition. As the various fungal associates have different temperature optima, they can influence the performance of *I. typographus* differently under changing environmental conditions. Finally, we discuss why effects of drought on tree-killing by bark beetles are still poorly understood and provide an outlook on future research on this eruptive species using both, field and laboratory experiments.

## Key message

- Drought-induced mass outbreaks of the bark beetle *Ips typographus* cause extensive tree mortality in European Norway spruce forests.
- Altered tree chemistry due to heat and drought affects composition, amounts, and emission rates of specialised metabolites including defence compounds.
- Specialised metabolites have positive and negative impacts on bark beetle host aggregation and acceptance on spatial and temporal scales.
- Drought can affect the performance of *I. typographus* and also its symbiotic ophiostomatoid fungi that detoxify tree defence compounds and produce beetle semiochemicals.

## Introduction

The Eurasian spruce bark beetle, *Ips typographus* (L.) (Coleoptera: Curculionidae: Scolytinae), is the most destructive forest pest of Norway spruce, *Picea abies* (L.) Karst. (Pinales: Pinaceae). It is one of the few bark beetle species worldwide that can undergo eruptive population outbreaks and lead to extensive forest mortality (Bentz et al. 2019; Kausrud et al. 2012). Rapid population growth commonly follows abiotic disturbance events, such as wind throw, snow break, high temperatures and drought (Jakoby et al. 2019; Marini et al. 2017; Seidl et al. 2016; Stadelmann et al. 2014). Climate change has caused an increased frequency and severity of hot and dry periods, thereby altering the distribution of trees and their susceptibility to diverse abiotic and biotic stressors (Allen et al. 2010; Jactel et al. 2012; Rouault et al. 2006). A number of recent articles have addressed the effects of abiotic factors on tree mortality and defence (Berini et al. 2018; Hartmann et al. 2018; Holopainen et al. 2018) and drought-related interactions between bark beetles and trees, especially *Dendroctonus* species attacking *Pinus* hosts in North America (Kolb et al. 2016, 2019; Sambaraju et al. 2019). Knowledge of climate change effects on Norway spruce defence chemistry, *I. typographus* population dynamics and interactions with associated organisms such as ophiostomatoid fungi (Kandasamy et al. 2019; Kirisits 2004; Zhao et al. 2019b) is crucial to understand the life history processes of this insect and also provides the basis for reliable predictions of mass outbreaks (Biedermann et al. 2019; Huang et al. 2020).

Here, we review chemo-ecological, molecular and behavioural aspects of this multitrophic system from the perspectives of bark beetles, symbiotic fungi and Norway spruce. In drought-stressed conifers, the decline of photosynthetic activity due to closure of stomata results in reduced availability of carbon for primary and secondary metabolism. In particular, drought stress affects the investment of carbon into plant growth and the maintenance of life-sustaining mechanisms such as respiration and defence (Adams et al. 2013; McDowell et al. 2008). Drought stress can have negative, neutral or even positive effects on constitutive (preformed) and induced tree defences, depending on the intensity and frequency of the triggering event and recovery times (Ayres and Lombardero 2000; Eyles et al. 2010; Koricheva et al. 1998; Niinemets 2010). Apart from acute stress events, stress history and tree genotype mediate drought effects on tree resistance and defence (Lopez-Goldar et al. 2018). Resin flow, for example, which most effectively protects Pinaceae against biotic invaders, is highly variable among trees and was shown to increase with mild to moderate drought but to decrease and even stop with severe water deficiency (Gaylord et al. 2013; Lieutier 2004; Lombardero et al. 2000; Netherer et al. 2015). Moreover, drought can impair further induction of defences such as formation of traumatic resin ducts in response to physical injury, insect and/or fungus attack (Klutsch et al. 2017). Conifer resin contains mainly terpenes; their concentration and emission rates are strongly influenced by heat and drought in both positive and negative ways (Ferrenberg et al. 2017; Holopainen et al. 2018; Mattson and Haak 1987). In the following sections, we focus on biochemical processes in drought-stressed trees with particular focus on specialised (secondary) metabolite concentrations, emission of volatile organic compounds (VOC) (Table 1) and their role as essential cues for *I. typographus* host selection, aggregation and host acceptance.

**Table 1:** Abbreviations used in manuscript, figures and table

Abbreviation	Term
ABA	Abscisic acid
GR	Gustatory receptors
IR	Ionotropic receptors
MeJA	Methyl jasmonate
NSC	Non-structural carbohydrates
OR	Odorant receptors
OSN	Olfactory sensory neurons
PP	Polyphenolic paremchyma cells
VOC	Volatile organic compounds

The diversity of specialised metabolites comprises terpenoids, phenolics and some compounds derived from fatty acids and proteins. Proteins, for instance, are involved in many stress-related processes such as the strengthening of plant cell walls (Fossdal et al. 2007). Concentration and composition of specialised metabolites determine the bark quality for insect and fungal performance (Erbilgin et al. 2017; Franceschi et al. 2005; Holopainen et al. 2018; Månsson 2005). A healthy tree has constitutive defence traits in the form of terpene-rich resins stored in specialised resin ducts or canals and (poly)phenolic compounds, produced and stored in polyphenolic parenchyma (PP) cells (Franceschi et al. 2005). Constitutive phenolic diversity and specific phenolic compounds have been recognised to enhance tree resistance against ophiostomatoid fungi associated with bark beetles (Brignolas et al. 1998; Evensen et al. 2000). However, induced defence reactions involving the upregulation of specialised metabolites such as monoterpenes and phenolics in response to wounding, fungal attack or methyl jasmonate application are more reliable to indicate the potential of a tree to defend itself (Balogh et al. 2018; Martin et al. 2003; Schiebe et al. 2012; Zhao et al. 2010, 2011b). Methyl jasmonate (MeJA) is an organic compound derived from the plant hormone jasmonic acid that induces plants to produce multiple types of defence chemicals. By activating enzymes involved in terpene synthesis (Celedon and Bohlmann 2019), MeJA treatments can lead to increased toxic terpene levels in tree tissue (Zeneli et al. 2006). Such tree reactions potentially impact host acceptance and colonisation success of *I. typographus*, which we discuss in Sect. 3.

Bark defence compounds affect bark beetle host acceptance for feeding and oviposition. These compounds indicate host quality via taste of *I. typographus* or provide olfactory signals from host and non-host trees (Byers et al. 1998; Faccoli et al. 2005; Zhang and Schlyter 2004). During beetle dispersal, emergence and flight are not directly dependent on chemical cues, but mainly driven by temperature and wind conditions (Byers 2000). Flight exercise and fat loss during dispersal stimulate response of bark beetles to kairomones and pheromones (Byers et al. 1998; Graham 1959; Wijerathna and Evenden 2019), and beetles become sensitive to cues from suitable habitats or individual trees. Although host tree distribution in the landscape is unpredictable and patchy (Kausrud et al. 2011), *Ips* pioneer beetles appear to select spruce trees of suitable age, nutritional value and defence status with remarkable precision (Wood 1972). The exact mechanisms for choosing susceptible trees, such as drought-stressed or wind-thrown individuals, are as yet little known. The role of volatile cues from stressed host trees for host-seeking pioneer beetles remains elusive. Alternatively, beetles are supposed to land randomly (Byers 1989, 1999) and select hosts based on chemical stimuli acting at short distances. Navigation of conspecifics to suitable hosts are then guided by pheromones and may be further supported by volatiles released by symbiotic fungi (Kandasamy et al. 2016). Associated ophiostomatoid fungi produce pheromone components and help to exhaust tree defences. Fungal symbionts are able to detoxify terpenes and phenolics (Lieutier et al. 2009; Wadke et al. 2016; Wang et al. 2014; Zhao et al. 2019a). Understanding these multipartite relationships is essential to predict *I. typographus* performance and is facilitated by the now available genome of this species (Powell et al. 2020).

## The Norway spruce perspective

### Tree metabolism under drought stress

**Fig. 1:**
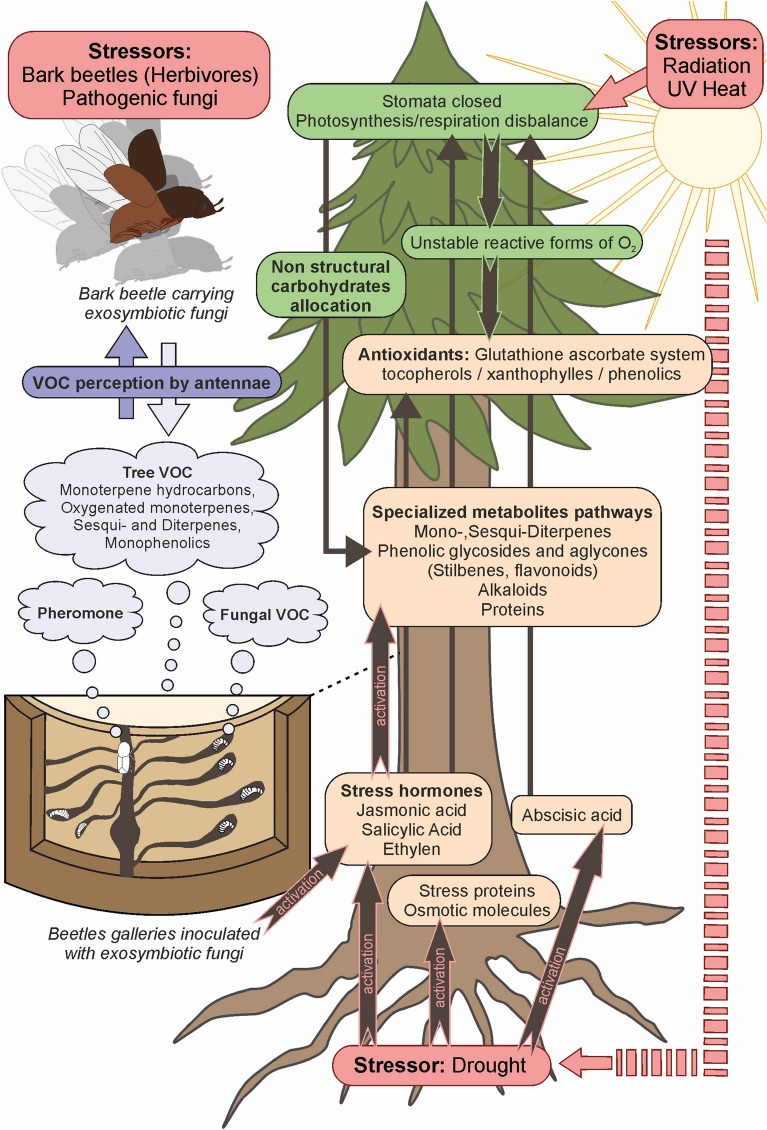
Conceptual scheme of interactions among Norway spruce, *Ips typographus* and symbiotic ophiostomatoid fungi under drought conditions. Stressors such as heat and drought activate stress hormones and stress proteins and trigger the closure of stomata via activation of abscisic acid. Stomatal closure and reduced photosynthetic activity cause a cascade of biochemical reactions in the tree involving pathways for the biosynthesis of antioxidants and specialised metabolites from non-structural carbohydrates. Activation of hormones and proteins as well as biochemical reactions are indicated by brown arrows. Defence compounds in the bark and volatile organic compounds (VOC) such as monoterpene hydrocarbons and oxygenated monoterpenes as well as pheromone components (fair blue clouds) are important gustatory and olfactory signals for host searching and attacking bark beetles. Associated fungi play a crucial role in detoxification of compounds and attraction of beetles to the tree

Conifer defences have evolved under multifaceted abiotic and biotic selection pressures for millions of years. Expression of defence mechanisms differs substantially among tree species and underlies genotypic, phenotypic, as well as ontogenetic variation. Tree defence is strongly shaped by environmental conditions, such as light and temperature (Berini et al. 2018; Ferrenberg et al. 2017). Heat and drought conditions continuously reduce water tension in the xylem. Tree species with mainly isohydric behaviour, such as many Pinaceae, do not tolerate water potential to drop below a certain threshold and respond by closing their stomata to prevent water loss (Fig. 1) (Moran et al. 2017; Sevanto et al. 2014). Soil water supply and climatic parameters impact tree net-photosynthesis and consequently the supply of non-structural carbohydrates (NSC), mainly low-molecular weight sugars and starch (Adams et al. 2013; Hoch et al. 2003). NSC pools, which vary among plant tissues and fluctuate over the season, are the central energy resources of a tree for respiration, growth, reproduction, storage, as well as constitutive and induced production of specialised metabolites (Fig. 1) (Bansal and Germino 2009; Hoch et al. 2003; Körner 2003). Availability and allocation of NSC to defence change under drought conditions in a nonlinear way depending on compound class, tree characteristics, affected plant organs, presence of biotic agents, timing and intensity of stress (Huang et al. 2020). Under moderate drought, conifer trees tend to sustain or even reinforce defences against attacks (Ferrenberg et al. 2015; Jacquet et al. 2014; McDowell and Sevanto 2010; McDowell 2011). There is increasing evidence that trees shunt NSC to specialised metabolite biosynthesis and repair stress-induced damages even when they are severely stressed (Hartmann et al. 2018; Huang et al. 2019) rather than simply trading off growth against defence (Bryant et al. 1983; Herms and Mattson 1992). The ability of conifer trees to mobilise defences might be impaired by multiple stressors, in particular when biotic and abiotic factors co-occur. For example, water deficit mainly reduced accumulation of monoterpenes in necrotic lesions produced after inoculation of the fungus *Grosmannia clavigera* in *Pinus banksiana* and *Pinus contorta*, pointing to an increased susceptibility of drought-stressed seedlings and trees to biotic attack (Hussain et al. 2020; Klutsch et al. 2017; Lusebrink et al. 2016). In conifers, terpene synthesis and emission of volatile compounds have been documented to rise with increasing water stress, but to eventually decrease under severe drought (Holopainen et al. 2018; Lieutier 2004). Such a response underlines the relevance of terpenes as potential stress markers indicating changes in tree susceptibility to biotic attack. In conifer trees, bark contents of polyphenols have not shown such trends, while endogenous levels of phenolic compounds are increased under drought stress in other plants such as rapeseed *Brassica napus* or thyme *Thymus vulgaris* by activation of the phenylpropanoid metabolism (Sharma et al. 2019). Interestingly, due to variations in stress-mediated responses on bio-synthetic pathways and trade-offs between defence compounds, total amounts of numerous specialised compound classes in the bark (but not terpenes) often remain unaffected by drought and therefore do not reliably indicate stress (Koricheva et al. 1998).

### Biochemical stress indicators in Norway spruce

From the biochemical perspective, tree response to drought stress is a complex mechanism. It involves universal signatures present in all plants, but also includes specific pathways found only in certain taxa. When trees experience a lack of soil water, expression of genes related to certain proteins and plant hormones such as abscisic acid (ABA) but also jasmonic acid, salicylic acid and ethylene is upregulated (Eldhuset et al. 2013; Jackson et al. 1995; Tan and Blake 2006) (Fig. 1). Initiation of signal cascades by shifts in gene expression patterns already happens in the early stage of a drought event promoting the closure of stomata by ABA (Moran et al. 2017). Reduced stomatal conductance results in a decrease in CO_2_ in chloroplasts, triggering oxidative and photo-oxidative stress. A disturbed redox state of cells can lead to damages of cell components caused by free radicals and peroxides, i.e. the active forms of oxygen (Felicijan et al. 2015). In return, trees start to synthesise antioxidants such as tocopherols (vitamin E) and xanthophyles (carotinoids) to quench reactive oxygen species (Tausz et al. 2001). The main pathway for removal of reactive oxygen species is the ascorbate–glutathione cycle. Key compounds of this cycle are ascorbate (ascorbic acid or vitamin C), which is the most abundant water-soluble antioxidant in plant cells, and glutathione, essential for the regeneration of ascorbate (Urbanek Krajnc et al. 2014). Ascorbate and glutathione levels increase in Norway spruce needles during mild drought stress, along with a changed ratio of reduced and oxidised glutathione (Tausz et al. 2004, 2001). A rise in glutathione and cysteine levels was also observed in response to salicylic acid application on bark sections of Norway spruce. Such treatment was shown to reduce the number of spruce bark beetle entrance holes and maternal gallery lengths compared to untreated controls by increasing expression levels of defence-related genes via systemic acquired resistance (Urbanek Krajnc et al. 2011). Apart from anti-oxidative defence and redox control, glutathione plays multiple roles in plant metabolism, promoting resistance to various pathogens (Tausz et al. 2004). Experimental infections of Norway spruce bark with ophiostomatoid fungi revealed interactions between the ascorbate–glutathione system and chemical defence via phenolic compounds. Antioxidants such as polyphenols are oxidised to phenoxyl radicals, which are then reduced by ascorbate to avoid DNA damage of trees (Hammerbacher et al. 2019; Urbanek Krajnc et al. 2014). The synthesis and storage of phenolics in PP cells, in combination with the ascorbate–glutathione cycle, have been increasingly recognised as pivotal systems for tree response to biotic and abiotic stress (Felicijan et al. 2015). The importance of particular phenol families and specific phenolic compounds for tree resistance is clearly demonstrated by their effects on *I. typographus* and associated blue-stain fungi, as described in Sects. 3, 4. Several other biochemical and tree physiological characteristics are considered to be indicators of tree stress such as contents of plant stress hormones (Fig. 1), changes in proteome, concentrations of key proteins, photosynthetic ability (chlorophyll fluorescence, stomatal conductance), fluctuations in monoterpene contents (Kainulainen et al. 1992) and emissions of volatiles (Holopainen et al. 2018).

### Constitutive terpene patterns in Norway spruce

**Fig. 2:**
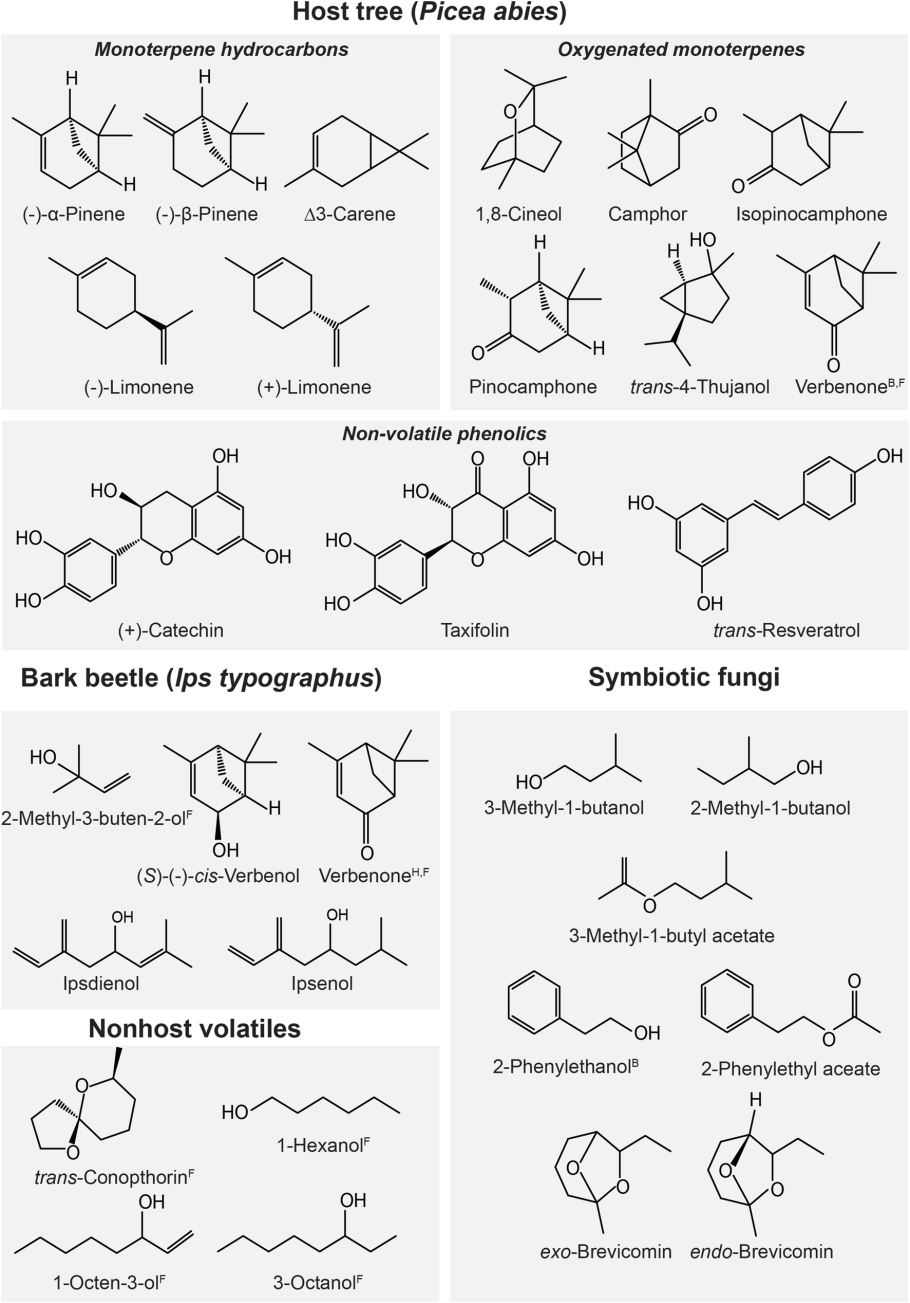
Chemical structures of some selected compounds that mediate interactions among Norway spruce trees, bark beetles and fungi. The compounds are divided into four major groups based on their origin: Host tree (*Picea abies*)—monoterpene hydrocarbons, oxygenated monoterpenes and non-volatile phenolics; bark beetle (*Ips typographus*) pheromones; nonhost tree volatiles; symbiotic fungal volatiles that function as insect semiochemicals. See Table 2 for more details on the ecology of individual compounds. Several bark beetle semiochemicals have multiple origins, and other known sources are denoted as *H*: host tree or *B*: beetle or *F*: fungus in suffixes next to compound names

The composition of terpenoids in Norway spruce bark is strongly influenced by environmental conditions, tree genotype and tissue types (Schönwitz et al. 1990). Moreover, observed terpene blends depend on the mode of extraction with differences between volatiles that are directly absorbed from the bark surface or extracted from the phloem using organic solvents (Hietz and Baier 2005). Table 2 and associated Fig. 2 list terpenoid compounds typically found in Norway spruce and relevant information regarding tree defence, host recognition and attack success of *I. typographus* as well as interactions with fungal symbionts. Extraction of monoterpene hydrocarbons from spruce phloem has revealed considerable variability in the proportion of different compounds with α-pinene (23–39%), β-pinene (25–58%), β-phellandrene (5–19%), limonene (1.5–4%), myrcene (1.6–3.4%), Δ-3-carene (0.6–1.1%) and camphene (0.2–1.1%) predominating in the low molecular fraction. This variability is likely due to differences in the age of sampled trees (Baier et al. 2002; Borg-Karlson et al. 1993; Silvestrini et al. 2004). Furthermore, proportions of (-)-enantiomers of chiral compounds (Borg-Karlson et al. 1993; Lindström et al. 1989; Persson et al. 1996) differ with genetic origin (Kännaste et al. 2012), plant tissue (Borg-Karlson et al. 1993), as well as among and even within geographic areas (Persson et al., 1996). Sesquiterpenes such as *α*-longipinene, *β*-farnesene and germacrene D occur in much smaller fractions in spruce bark than monoterpenes (Schiebe et al. 2012; Zeneli et al. 2006; Zhao et al. 2011b). Diterpenes (resin acids, alcohols and hydrocarbons) such as sandaracopimarate, abienol and thunbergol are less volatile but have been found to correlate with tree resistance against *I. typographus* and associated fungi (Zeneli et al. 2006; Zhao et al. 2011b). Shifts in terpene blends and concentrations are strongly linked to tree stress and interactions with bark beetles and their fungal symbionts. In healthy trees, oxygenated monoterpenes are represented only in trace amounts. However, after bark beetle attack and fungal inoculation, the concentration of oxygenated monoterpenes gradually increases via detoxification of monoterpene hydrocarbons in the beetle gut and by symbiotic fungi (Kandasamy et al. 2016; Leufvén and Birgersson 1987; Petterson and Boland 2003; Schiebe et al. 2019).

**Table 2:** Ecologically relevant chemical compounds that mediate interactions among Norway spruce, *Ips typographus* (IT), and ophiostomatoid fungi

Compounds and their origin	Physiological changes to host tree chemicals in^a^			Bark beetle (*Ips typographus*)	Impact on fungal growth/ Fungal origin^d^
	Response to MeJA	Response to fungi	Antennal response^b^	Ecological relevance/Tree physiological response^c^	
*Tree/Monoterpene hydrocarbons*^*4,10,26,27,28,29,30,32,33,38,39,43,47*^					
*α*-Pinene	↑abs^27,46^	↓abs^46^	GC-EAD^21,33^, SSR^3,33^	Host recognition?^1^, conifer habitat cue?^41^	Delay and inhibition of fungal growth in vitro, *Ep*^28^
(−)-*α*-Pinene	↑abs^32^ no^46^	↑abs^4^ no^43,46^	GC-EAD^21,33^, SSR^1,3,33^	Pheromone precursor^31^ Attraction (with pheromone)^15^ higher proportion in surviving, lower in killed trees (?)^4,32,46^	–
*β*-Phellandrene	↑abs^27,46^ ↓rel^43^	↑abs^46^	GC-EAD^2,21,33^	–	–
(−)-*β*-Pinene	↑abs^32,46^	↑abs^4^ no^46^	GC-EAD^21,33^, SSR^3,33^	–	Delay and inhibition of fungal growth in vitro, *Ep*^28^
Δ3-Carene	↑abs^4,46^	↑abs^4,28,46^ ↑rel^43^	GC-EAD^21,33^, SSR^1,3,33^	↓abs in IT killed trees (MeJA)^32^	Delay and inhibition of fungal growth in vitro, *Ep*^28^
(−)-Limonene	↑abs^4,46^	no^46^	GC-EAD^21,33^, SSR^3,33^	↑abs and ↑rel in bark of less attractive and surviving trees; abs↓ and ↓rel in IT killed trees^32,46^	–
(+)-Limonene	↑abs^4,46^	↓abs^4,46^	GC-EAD^21,33^, SSR^3,33^	↓abs in IT killed trees^32^	Delay and inhibition of fungal growth in vitro, *Ep*^28^
*Tree/Oxygenated monoterpenes*^*4,26,27,28,30,32,33,36,43,47*^					
1,8-Cineole (Eucalyptol)	↑abs^27,32^ ↑rel^33,43^	↑abs^4^	GC-EAD^2,21,33^, SSR^2,3^	Antiattractant^2,6^, true resistance marker for tree survival^32^ ↑abs emission in IT attacked trees^33^; ↑abs in bark of IT surviving trees, ↓abs in IT killed trees^32^	–
Camphor	no^33^	–	GC-EAD^21,33^, SSR^33^	–	–
Pinocamphone	↑rel^33^	–	GC-EAD^21,33^	↑abs emission in IT attacked trees^33^	–
*trans*-Pinocarveol	–	↑abs^28^	–	–	Produced by yeasts^25^
*α*-Terpineol	↑rel^33^	↑abs^4^ ↑rel^4^	GC-EAD^2,33^, SSR^33^	–	Produced by yeasts^25^
trans-4-Thujanol (Sabinene hydrate)	–	–	GC-EAD^33^, SSR^33^	Antiattractant^9^ ↓abs with tree age^9^	–
(−)-Terpinen-4-ol	no^33^	↑abs^28^	GC-EAD^21, 33^ SSR^33^	–	Produced by yeasts^25^
Verbenone	–	–	GC-EAD^21,33^, SSR^3,33^	Antiattractant pheromone^6,35^; terminates aggregation of IT^33,35^ ↑abs emission in IT attacked and decomposed wood^8,33^	Produced by tree microbiota, ophiostomatoid fungi and yeasts^13,23,25^
*Tree/Phenolics*^*11,12,16,18,19,20,21,26,30,32,33,39*^					
(+)-Catechin	–	↑abs in lesions^11,12,16,20,26^	–	Antifeedant^17^ or anti-nutritive^19^ Marker of resistance to fungi (?)^11,12^	Fungal growth inhibitor^16^; synergizes with taxifolin^19^ Degradation by *Gp* and *Ge*^45^
Estragole (4-Allylanisole)	↑abs^32^	–	GC-EAD^2,33^, SSR^33^	↑abs emission in felled IT attacked trees^33^; ↓abs in IT killed, ↑abs in surviving trees/MeJA^32^	–
Gallocatechin	–	↑abs^20^	–	–	Inhibits growth and melanin biosynthesis in *Ep*^18,20^
*trans-*Resveratrol (Resveratrol)	–	↑abs^16^	–	Antifeedant for males^17^	abs↑in spruce clone more susceptible to *Ep*^16^
Taxifolin	–	↑abs in lesions^16,18^	–	Antifeedant^17,19^ and anti-nutritive^19^	Fungal growth inhibitor^11^; synergises with catechin^18,20^
*Beetle pheromones*					
2-Methyl-3-buten-2-ol (MB)	–	–	GC-EAD^2,14^, SSR^3^	Main pheromone component, produced by beetles *de novo*^5,8,34^	Produced by *Ge* and *Gp* de novo^42,44^
(*S*)-(−)-*cis*-Verbenol	–	–	GC-EAD^14^, SSR^3^	Aggregation pheromone component produced from α-pinene^5,8,31,34^	ND
(*S*)-(−)-Verbenone	–	–	GC-EAD^21,33^, SSR^3,33^	Terminates aggregation on host tree^33,35^	Produced by IT yeasts from *cis*-verbenol^23,26,25^
(*R*)-(−)-Ipsdienol	–	–	GC-EAD^14^, SSR^3^	Produced de novo by males after mating with females, weakly boosts the attraction of pheromone mixture MB/cis-Verbenol^7,34^	ND
(*R*)-(+)-Ipsenol	–	–	GC-EAD, SSR^3,33^	Produced de novo by males after mating with females, relict of ipsdienol^7,34,35^	ND
*Non-host tree volatiles*					
*trans-*Conophthorin	–	–	GC-EAD, SSR^3^	Angiosperm bark^41^, part of non-host volatiles anti-attractants blend^40,41^, antifeedant^17^	Produced by *Gp,Ge* de novo^44^
1-Octen-3-ol	–	–	GC-EAD, SSR^3^	Angiosperm bark^41^, part of non-host volatiles anti-attractants blend^40,41^	Produced by *Op* de novo^22^
3-Octanol	–	–	GC-EAD, SSR^3^	Angiosperm bark^41^, part of non-host volatiles anti-attractants blend^40,41^	ND
1-Hexanol	–	–	GC-EAD, SSR^3^	Angiosperm leafs^41^ (green leaf volatiles) blend component; part of non-host volatiles anti-attractants blend^40,41^	Produced by *Ge, Ob* and *Op* de novo^22^
(*E*)-2-Hexenol			GC-EAD, SSR^3^	Angiosperm leafs^41^ (green leaf volatiles) blend component; part of non-host volatiles anti-attractants blend^40,41^	–
(*Z*)-3-Hexenol			GC-EAD, SSR^3^	Angiosperm leafs^41^ (green leaf volatiles) blend component, part of non-host volatiles anti-attractants blend^40,41^	–
*Fungal compounds as bark beetle semiochemicals*					
*exo*- and *endo*-Brevicomin	–	–	GC-EAD^37^, SSR^3^	Increased attraction to pheromone blend^38^	Produced by *Gp, Ge, Ob*, *Op* de novo^44^
3-Methyl-1-butanol	–	–	SSR^22^	Component of short-range attraction blend in lab assay^22^	Produced by *Ep, Gp, Ge, Ob, Op* de novo^22^
2-Methyl-1-butanol	–	–	SSR^22^	Component of short-range attraction blend in lab assay^22^	Produced by *Ep, Gp, Ge, Ob, Op* de novo^22^
3-Methyl-1-butyl acetate	–	–	SSR^22^	Component of short-range attraction blend in lab assay^22^	Produced by *Ep, Gp* de novo^22^
2-Phenylethanol	–	–	SSR^22,33,37^	Produced also by males^7^, not field active^34^, component of short-range attraction in lab assay^22^	Produced by *Ep, Gp, Ge, Ob, Op* de novo^22^
2-Phenylethyl acetate	–	–	SSR^22^	Component of short-range attraction blend in lab assay^22^	Produced by *Ep, Gp* de novo^22^

Compounds were grouped based on their origin (tree, beetle, non-hosts and fungi/yeasts)

Tree compounds are further divided into monoterpene hydrocarbons, oxygenated monoterpenes and phenolics

The literature references are indicated by superscript numbers in the table. References corresponding to numbers in the table are listed in the footnote

*Ep: Endoconidiophora polonica*, *Gp*: *Grosmannia penicillata*, *Ge*: *Grosmannia europhioides*, *Ob*: *Ophiostoma bicolor*, *Op*: *Ophiostoma piceae*, ND: not detected, –: unknown or not tested

^a^Physiological changes to host tree chemicals in Norway spruce in response to MeJA application and inoculation with pathogenic fungi; arrows indicate absolute (abs) or relative (rel) decrease (↓) or increase (↑) compared to control treatments

^b^Perception of chemical compounds on *Ips typographus* antennae confirmed either by electroantennography (GC-EAD) or single sensillum recording (SSR) or both

^c^Ecological relevance of host chemicals to bark beetles and tree’s physiological response upon bark beetle infestation

^d^Impact of host tree chemicals on fungal growth and fungal origin of beetle semiochemicals

*References:* Andersson (2012)^1^; Andersson et al. (2010)^2^; Andersson et al. (2009)^3^; Baier et al. (2002)^4^; Bakke (1976)^5^; Binyameen et al. (2014)^6^; Birgersson et al. (1984)^7^; Birgersson and Bergström (1989)^8^; Blažytė-Čereškienė et al. (2015)^9^; Borg-Karlson et al. (1993)^10^; Brignolas et al. (1995)^11^; Brignolas et al. (1998)^12^; Cale et al. (2019)^13^; Dickens (1981)^14^; Erbilgin et al. (2007)^15^; Evensen et al. (2000)^16^; Faccoli and Schlyter (2007)^17^; Hammerbacher et al. (2019)^18^; Hammerbacher et al. (2014)^19^; Hammerbacher et al. (2018)^20^; Kalinová et al. (2014)^21^; Kandasamy et al. (2019)^22^; Leufvén et al. (1984)^23^; Leufvén and Birgersson (1987)^26^; Leufvén et al. (1988)^25^; Lieutier et al. (2003)^26^; Martin et al. (2002)^27^; Novak et al. (2013)^28^; Persson et al. (1996)^29^; Petterson and Boland (2003)^30^; Renwick et al. (1976)^31^; Schiebe et al. (2012)^32^; Schiebe et al. (2019)^33^; Schlyter et al. (1987a)^34^; Schlyter et al. (1989)^35^; Silvestrini et al. (2004)^36^; Tømmerås (1985)^37^; Tømmerås and Mustaparta (1984)^38^; Zeneli et al. (2006)^39^; Zhang and Schlyter (2003)^40^; Zhang and Schlyter (2004)^41^; Zhao et al. (2015)^42^; Zhao et al. (2011a)^43^; Zhao et al. (2019a)^44^; Zhao et al. (2019b)^45^; Zhao et al. (2010)^46^; Zhao et al. (2011b)^47^

### Stress-induced tree responses

Changes in Norway spruce bark chemistry, including the increased oxygenation of monoterpene hydrocarbons, have been clearly linked to biotic stress caused by *I. typographus* attack and fungus inoculation, or activation of induced tree defence by MeJA treatment (Martin et al. 2003; Novak et al. 2013; Schiebe et al. 2019). Oxygenated monoterpenes in conifer bark are ecologically significant as pheromone components of numerous bark beetle species (Francke and Vité 1983; Schlyter et al. 1989). Interconversion of verbenone from verbenols by yeasts and ophiostomatoid fungi growing in bark beetle brood galleries (Birgersson and Bergström 1989; Cale et al. 2019; Davis 2015) and potential stress-related biochemical regulation of oxygenated monoterpenes by trees suggests dual roles of these compounds in beetle communication and induced tree defence (compare Sects. 3 and 4). Heavily infested trees are no more attractive for spruce bark beetles. In *I. typographus* galleries*,* a large number of oxygenated compounds including camphor, α-terpineol, terpinene-4-ol and borneol were found (Leufvén and Birgersson 1987). The oxygenated monoterpene *trans*-4-thujanol shows repellent effects on both sexes of *I. typographus* and strongly decreases in concentration from saplings to mature Norway spruce (Blažytė-Čereškienė et al. 2015), rendering trees of larger diameter size more susceptible to infestation (Table 2).

Linking tree disposition to bark beetle attack to specific volatiles is challenging due to high heterogeneity among tree individuals and influence of temperature, light, and humidity on volatile emission rates (Hietz and Baier 2005; Santos et al. 2006; Zhang and Schlyter 2004). Although enhanced attraction of bark beetles to increased release rates of volatiles from spruce logs cannot be entirely excluded, evidence from the literature is limited as effects of VOC emissions and *I. typographus* attack have been rarely studied on healthy, mature trees. Most analyses of terpene contents and/or emissions in the context of drought focus on pine needles of mainly younger trees. For example, potted *Pinus pinaster* with moderate water deficits emitted more myrcene and less *β*-pinene from the canopy than control plants, but bark beetles (*Tomicus destruens*) performed worse in drought-stressed plants (Branco et al. 2010). Volatile emission rates from needles of drought-stressed *Pinus halepensis* seedlings significantly exceeded those of well-watered trees, but declined with continued drought (Ormeno et al. 2007). In the same tree species, drought stress also enhanced phloem terpene concentrations (Kelsey et al. 2014; Ormeno et al. 2007). In needles of severely drought-stressed Scots pine, and Norway spruce seedlings and saplings, total and individual monoterpene contents (especially *α*-pinene, limonene, tricyclene and camphene), and sesquiterpene contents increased significantly, while resin acids showed a decreasing trend (Kainulainen et al. 1992; Sancho-Knapik et al. 2017; Turtola et al. 2003). Sancho-Knapik et al. (2017) recently showed that accumulation of terpenes in mildly drought-stressed *Pinus sylvestris* coincided with stomatal closure and increase in ABA. Similarly, MeJA application enhanced the expression of terpene synthases regulating de novo synthesis of terpenes in Norway spruce needles (Martin et al. 2003). While these results seem to overall corroborate that increased terpene concentrations and emissions are triggered by moderate drought, monoterpene biosynthetic capacity might be reduced under severe drought-stress as demonstrated in saplings of *Abies grandis* (Lewinsohn et al. 1993).

## The *I. typographus* perspective: host selection and acceptance

### Olfaction and taste

**Fig. 3:**
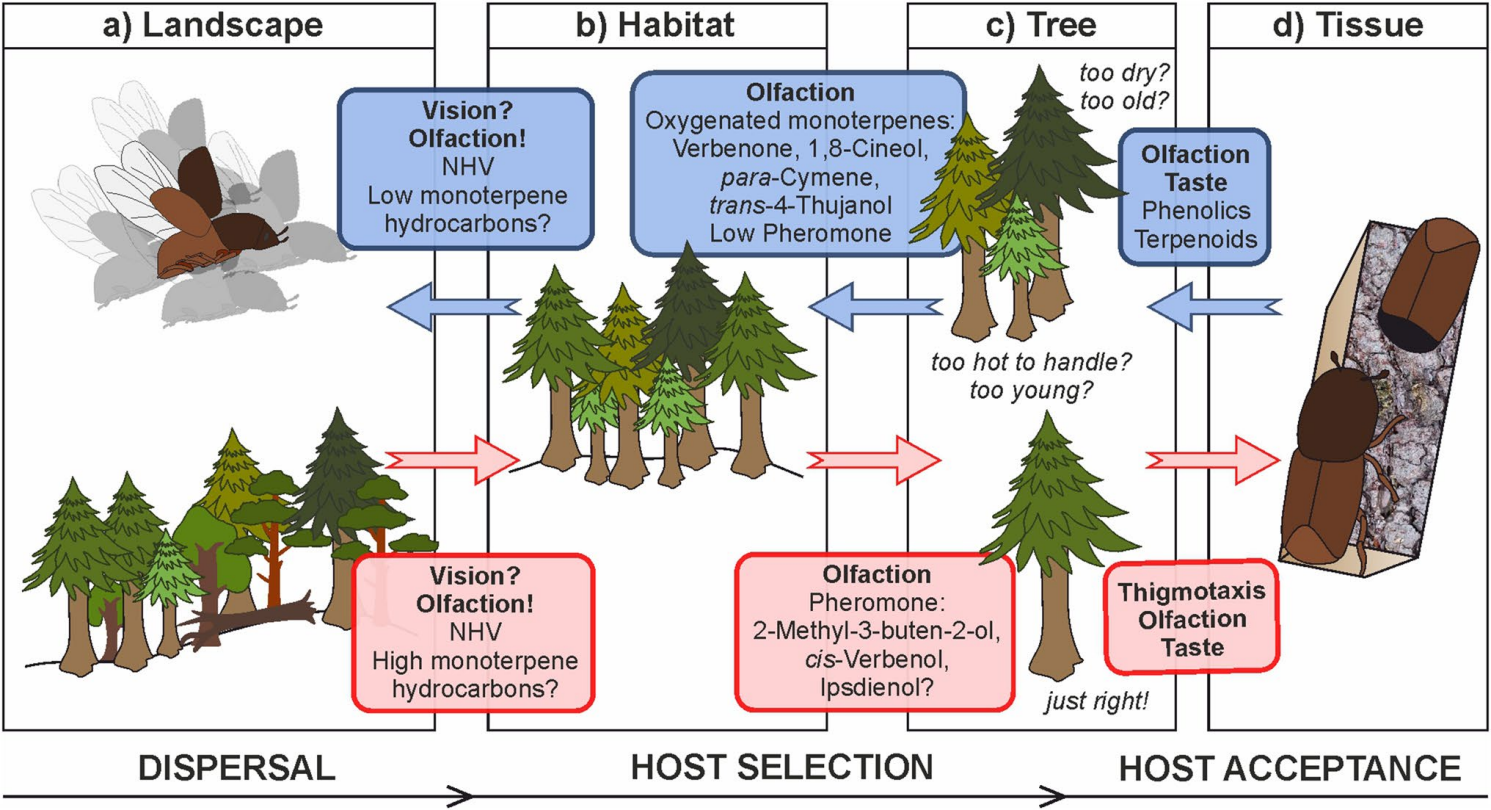
Behavioural sequence for *Ips typographus* in **a** landscape (dispersal), **b** habitat and **c** tree (both host selection), and **d** tissue (host acceptance) by positive (fair blue arrows and boxes) and negative cues (red arrows and boxes). Focus is set on the pioneering male beetles, whose rapidly produced pheromone signals guide the vast majority of both males and females to aggregate. The individual beetle follows a sequence of steps, guided by visual, chemo-sensory and thigmotactic cues

Bark beetles live in a complex, multitrophic environment. Their ability to survive depends on their senses, which mediate intra- and interspecific interactions with plants, animals, fungi and other microorganisms. Phytophagous insects discriminate host from non-host odours based on specific blends of compounds (Bruce and Pickett 2011). The volatile mixture released from newly cut Norway spruce logs was not found to particularly attract *I. typographus* in field trapping experiments (Lindelöw et al. 1992). In addition, neither individual host compounds of Norway spruce have been shown attractive (Schlyter and Birgersson 1999), nor did host material supplemented with synthetic aggregation pheromones increase trap catches (Schlyter et al. 1987). However, *I. typographus* has numerous sensory cells, specific for monoterpene hydrocarbons (Table 2), which might help to locate odours of suitable conifer habitats (Zhang and Schlyter 2004) and aid in close-range host acceptance after landing on trees (Fig. 3) (details see Sects. 3.2, 3.3).

Among the diversity of elaborate antennal structures in insects (Hansson and Stensmyr 2011), bark beetles have olfactory sensillae located merely on the ventral side of their club-shaped antennae. Olfactory sensory neurons (OSN) within olfactory sensillae (Hallberg 1982) show high specificity to many groups of semiochemicals, reflecting the major role of olfaction in bark beetle long- and short-range orientation (Fig. 3). Chemical signals are further processed in higher brain centres and eventually evaluated as positive and negative signals, which forms the basis for orientation towards or away from sources (Andersson et al. 2015). Host discrimination by insects in general is supported by particular co-localization of certain OSN types found among sensillar functional types (Andersson et al. 2010; Hansson and Anton 2000). In *I. typographus*, the most narrowly tuned OSN specifically respond to a single compound or few closely related molecules, while response spectra of generalist OSN comprise a broader range of plant volatiles (Andersson 2012). In several electrophysiological studies, 24 classes of OSN were identified for *I. typographus* (Andersson et al. 2009; Mustaparta et al. 1984; Schiebe et al. 2019; Tømmerås 1985). OSN responding to attractive or anti-attractive pheromonal compounds are highly specific, strongly reacting to only one or a few structurally related compounds (Andersson 2012; Andersson et al. 2009). Plant odour-responding OSN show a variety of response specificities, often quite specific to key ligands (1,8-cineole OSN) or more broadly tuned such as OSN for host monoterpene hydrocarbons (Table 2) (Andersson et al. 2009; Kandasamy et al. 2019; Schiebe et al. 2019). The specifity of OSNs likely reflects strong selection pressures on host recognition in *I. typographus*, making it such an efficient tree-killing bark beetle (Andersson et al. 2013).

In two transcriptomic studies on *I. typographus* antennae (Andersson et al. 2013; Yuvaraj et al. 2020), receptors for airborne molecules form three different multigene families: 73 odorant receptors (OR), 7 ionotropic receptors (IR) and 6 gustatory receptors (GR) were identified (Andersson et al. 2013; Yuvaraj et al. 2020). ORs detecting volatile compounds such as pheromones, plant odours and fungal volatiles have been investigated in *I. typographus* so far. IRs detecting nitrogen-containing compounds, aromatics, organic acids and temperature have not been investigated in detail yet. Only a fraction of olfactory receptors and their ligands that shape olfactory-driven behaviours in *I. typographus* are known. Recently, the first two *I. typographus* ORs tuned to single enantiomers of the bark beetle pheromone compounds ipsenol and ipsdienol have been functionally characterised (Yuvaraj et al. 2020) and their binding mechanisms described. The biological significance of the characterised ORs positions them as prime targets for pest control and use in biosensors to detect bark beetle infestations (Yuvaraj et al. 2020).

Compared to olfaction, taste perception of host phytochemicals in insects is much less explored (Pentzold et al. 2017) and knowledge is entirely lacking in bark beetles. Insects taste non-volatile plant metabolites via cuticular hairs, pegs, or pits abundant on the mouthparts, antennae, tarsi and genitalia. In bark beetles, the sense of taste might be very important during host selection (Elkinton and Wood 1980; Raffa et al. 2016) to discriminate host and non-host plants (Byers et al. 1998, 2000). Antennal transcriptomic investigations showed relatively low numbers of GR and taste-related IR in *I. typographus*. The same is true for *D. ponderosae* (Andersson et al. 2013), suggesting that taste receptors may be more expressed in mouth parts such as labella and less in antennae.

### Host selection

The behavioural sequence of host selection starts with dispersal through the landscape, ending in directed search of host trees in suitable habitats and final acceptance of host tissues (Fig. 3a–d). Although *I. typographus* is able to kill whole forest stands, identification of scattered, adequate hosts is challenging for a small insect. Mortality in the dispersal phase is high, with first-day mortality rates up to 50% (Coulson et al. 1980). At the habitat level (forest stands), olfactory cues keep bark beetles away from unsuitable sites (Fig. 3b) (Zhang and Schlyter 2004). Conifer bark beetles are guided by a combination of both positive cues (aggregation pheromone, host foliage monoterpene hydrocarbons, microbial volatiles) and negative cues (heterospecific pheromones, non-host volatiles, microbial volatiles) (Erbilgin et al. 2007; Franklin et al. 2000; Kandasamy et al. 2019; Raffa et al. 2016; Schlyter and Birgersson 1999; Zhang and Schlyter 2004). *Ips typographus* beetles avoided the source of non-host volatiles (six green-leaf alcohols, two bark alcohols and a ketal) (Table 2, Fig. 3b) (Zhang and Schlyter 2004), which are inhibitory to many bark beetle species. Having likely evolved in mixed forests landscapes (Lindgren and Raffa 2013), *I. typographus* is well adapted to avoid non-host trees. It is less clear how pioneer beetles locate particular susceptible hosts in semiochemically diverse habitats without any pheromone release.

The exact mechanisms of beetle orientation to single trees and final choice of suitable hosts based on their stress levels are elusive. Beetles seem to recognise both severely/chronically stressed trees and dying trees that are heavily attacked by other beetles (“*too dry, too old*”) as unsuitable hosts (Fig. 3c, top tree). In a field-bioassay, chronically/severely drought-stressed Norway spruce trees were less accepted for gallery initiation than vital but acutely drought-stressed ones; the cues are as yet unknown (Netherer et al. 2015). In trees at advanced stage of attack, after female egg deposition, males produce ipsdienol (*R*-(−)) and ipsenol (*R*-(+)) in their hindguts (Birgersson et al. 1984; Kohnle et al. 1991), with variable roles from attractants at low doses to repellents at higher doses (Schlyter et al. 1989). When nutritive resources are increasingly occupied by conspecific beetles and fungi, *cis*-verbenol is oxygenated to the corresponding ketone, verbenone by yeasts (Leufvén et al. 1984) and possibly by ophiostomatoid fungi (compare Sect. 4, Table 2) (Blomquist et al. 2010; Leufvén and Birgersson 1987). Increased emission of verbenone coincides with successful fungal establishment (Cale et al. 2019). Verbenone acts as anti-attractant at different doses alone (Bakke 1981), or in synergism with non-host volatiles (Zhang and Schlyter 2003) and counteracts attractive effects of aggregation pheromones (Binyameen et al. 2014; Lindgren and Miller 2019). Similarly, oxygenated monoterpenes such as 1,8-cineole released from heavily attacked trees show anti-attractant effects (Andersson et al. 2010). The compound 1,8-cineole was found to be the best predictor of resistant trees among several other specialised metabolites. The inducibility of 1,8-cineole, in turn, strongly increased the survival of trees from colonisation attempts by beetles (Schiebe et al. 2012).

Inhibitory effects of *trans*-4-thujanol in laboratory olfactometer experiments might explain low attraction of *I. typographus* to younger Norway spruce trees where this compound is more abundant (Blažytė-Čereškienė et al. 2015). Non-stressed trees (“*too hot to handle, too young*”) (Fig. 3c) are expected to be less susceptible and able to strongly induce defences during biotic attack as demonstrated using MeJA treatment (Zhao et al. 2011a). Intermediately stressed trees (“*just right*”) can be successfully attacked, and further arriving beetles emit aggregation pheromones rapidly upon landing. Direct effects of drought on pheromone synthesis are not known. The pheromone consists of the two active compounds 2-methyl-3-buten-2-ol and cis-verbenol (Bakke 1976; Schlyter et al. 1987). Most aggregation pheromones of *Ips* and *Dendroctonus* species are biosynthesised de novo (Byers and Birgersson 1990; Schlyter and Birgersson 1999), while some compounds are derived from host compounds (Blomquist et al. 2010; Tittiger and Blomquist 2017). Biosynthesis of *cis*-verbenol depends on the amount of (-)-*α*-pinene in the host bark (Lindström et al. 1989). During bark beetle attack, *α*-pinene is increasingly emitted from lacerated resin canals (Ghimire et al. 2016; Schiebe et al. 2019) and may attract more beetles in synergy with low amounts of pheromone (Erbilgin et al. 2007). When nuptial chambers are established by pioneer bark beetles at susceptible trees of reduced defences (Fig. 3c) maximum production of aggregation pheromone follows (Birgersson et al. 1984; Schlyter and Löfqvist 1986). Thus, *α*-pinene is a key compound for initial mass attack and semiochemicals production (Table 2).

### *Ips typographus* host acceptance and colonisation success

The chance of *I. typographus* to survive host colonisation attempts strongly depends on a tree’s response to the attack (Figs. 1 and 3d). Experimental inoculations with ophiostomatoid fungi and MeJA treatment induce immune responses involving changes in anatomical and chemical features of living bark and wood. These studies have revealed important mechanisms of tree defence such as the priming of inducible defences of Norway spruce for an accelerated and intensified response to future bark beetle attack (Krokene 2015; Mageroy et al. 2020). Induced formation of traumatic resin ducts and accumulation of terpenes in affected tissues reduce the number of *I. typographus* attacks, parental tunnel lengths and numbers of deposited eggs, as well as the growth of ophiostomatoid fungi (Erbilgin et al. 2006; Zeneli et al. 2006; Zhao et al. 2011a). Increased terpene concentrations triggered by MeJA application were reported to reduce or even prevent bark beetle attacks when exceeding a certain threshold (Zhao et al. 2011b).

Bark beetles, in turn, have adapted to tree defences by engaging in symbiotic interactions with fungi that detoxify defence compounds and help them to colonise even healthy trees (Lindgren and Raffa 2013). Yet, certain substances such as limonene, myrcene and Δ-3-carene are particularly toxic for beetles, limiting tunnelling and oviposition (Erbilgin et al. 2017; Everaerts et al. 1988; Wallin and Raffa 2002). In in vitro assays, limonene inhibited the growth of *Endoconidiophora polonica*, an important blue-stain fungal associate of *I. typographus* (Novak et al. 2013). Feeding of *I. typographus* on artificial diet was reduced when adding (+)-limonene (Faccoli et al. 2005), while (−)-limonene was linked to reduced attractiveness of Norway spruce for landing beetles (Zhao et al. 2010). However, the increase in oxygenated monoterpene 1,8-cineole in bark samples was the strongest predictor of induced defence in Norway spruce, leading to higher tree survival from natural attacks by *I. typographus* (Schiebe et al. 2012) (see previous section on anti-attractant activity and Table 2). In addition to 1,8-cineole, monoterpene hydrocarbons such as Δ-3-carene, myrcene, (-)-*α*-pinene, *β*-pinene and the diterpene thunbergol were found in increased amounts in the bark (Schiebe et al. 2012). These data support the assumption that enhanced monoterpene concentrations, as observed under moderate drought, increase tree defence against bark beetle attack. Apart from terpenoid compounds, fatty acids can be toxic to bark beetles (Erbilgin et al. 2017; Ishangulyyeva et al. 2016). Fatty acids are major components of lipids and involved in the synthesis of plant hormones. However, it is not known if *I. typographus* and its symbiotic fungi are influenced by fatty acids, and how fatty acid contents in bark are affected by drought.

A number of recent studies focused on the particular role of phenolic compounds in tree defence and effects on beetle-fungus interactions (Hammerbacher et al. 2019, 2014). Phenolics accumulate in hypersensitive wound reaction zones caused by pathogen attack (Christiansen et al. 1987; Krokene et al. 2001). Spruce phenolics are stored in PP cells (Franceschi et al. 2005), and the presence of phenylalanine ammonia lyase in these structures suggests that they are directly synthesised in these cells in response to biotic attack (Li et al. 2012). Phenylalanine ammonia lyase is an important enzyme of the phenylpropanoid pathway and catalyses the first step in the synthesis of polyphenolic stilbenes and flavonoids (Metsämuuronen and Sirén 2019). Stilbenes are known to protect the heartwood of pines from microbial decay and have nematocidal and fungicidal effects (Chong et al. 2009; Witzell and Martín 2008). The stilbenoids resveratrol and its glycosides piceid, astringin and isorhapontin are mainly present in Norway spruce phloem (Hammerbacher et al. 2011; Metsämuuronen and Sirén 2019). Resveratrol glycosides have been shown to accumulate in response to fungal infection and have antifungal activity (Evensen et al. 2000; Hammerbacher et al. 2011). Despite high stilbene levels, spruce trees suffer from fungal colonisation and fatal bark beetle infestation (Brignolas et al. 1995; Schiebe et al. 2012). This is attributed to the “fungus effect” (Brignolas et al. 1998) resulting in decreased stilbene glycosides around fungal infections (Viiri et al. 2001) due to degradation of phenolic glycosides to aglycones and use of these compounds for fungal metabolism (Hammerbacher et al. 2013). Highly virulent ophiostomatoid fungi are most efficient in degrading stilbenes and flavonoids (compare Sect. 4). The key virulence factor, catechol dioxygenase catalysing the first step in phenolic degradation, however, cannot degrade flavonols and dihydroflavonols (Hammerbacher et al. 2019; Wadke et al. 2016).

Activation of the flavonoid pathway, mainly the synthesis of proanthocyanidins, such as the flavan-3-ol monomers (+)-catechin and (+)-gallocatechin as well as condensed tannins, has been consistently linked to increased tree resistance to biotic attack (Brignolas et al. 1998; Hammerbacher et al. 2014, 2018; Lieutier et al. 2003) (Table 2). Flavan-3-ols and their metabolic precursor taxifolin showed anti-fungal effects in a synergistic way (Hammerbacher et al. 2019). Moreover, catechin- and taxifolin-enriched diet significantly reduced tunnel lengths and weight gain of *I. typographus* males in feeding bioassays (Faccoli and Schlyter 2007; Hammerbacher et al. 2019). Metabolites of taxifolin, i.e. laricitrin and its precursor myricetin, as well as the phenylpropene estragole (4-allylanisole) were induced significantly stronger in Norway spruce trees that eventually survived *I. typographus* attack (Schiebe et al. 2012). Furthermore, activation of flavanone synthases by jasmonic acid, salicylic acid and ABA (Fig. 1) has been linked to increased tolerance to biotic and abiotic stress, including drought (Felicijan et al. 2016; Hammerbacher et al. 2019; Nagy et al. 2004). So far, no clear relationships between drought and phenolics have been found (Holopainen et al. 2018). Much more research on the complex biochemical processes in conifers coupling anti-oxidative and insect/pathogen defence (Witzell and Martín 2008) is therefore needed.

## The ophiostomatoid fungus perspective

### Fungal symbionts of *I. typographus*

Microorganisms play important roles in the successful colonisation and reproduction of most conifer bark beetles. Many bark beetles have complex and dynamic multipartite interactions with bacteria, yeasts and fungi (Davis 2015; Popa et al. 2012; Six 2013) modulated by various factors such as temperature, humidity, moisture content of plant tissues, host phytochemistry and the presence of other interacting organisms in their galleries such as mites and nematodes (Biedermann et al. 2019). Among microbes, associations between bark beetles and fungi are relatively well studied (Franceschi et al. 2005; Kirisits 2004; Six 2012). The majority of fungal associates of bark beetles are ascomycetes, commonly known as ophiostomatoid or blue-stain fungi. *Ips typographus* has numerous fungal partners where compositions of fungal communities change with space and time (Chang et al. 2019; Linnakoski et al. 2012). Based on analyses of *I. typographus* fungal communities, the following species are considered to be the most frequent and most dominant symbionts: *E. polonica*, *Grosmannia penicillata*, *Ophiostoma bicolor*, *Grosmannia europhioides* (synonym *Grosmannia piceiperda*) and *Ophiostoma ainoae* (Kirisits 2004; Linnakoski et al. 2012). According to the ‘classical paradigm’, fungi are considered to support bark beetle attack by overcoming and exhausting the host tree defence system and aid beetles to promote tree death (Christiansen et al. 1987; Krokene 2015; Krokene et al. 2001; Lieutier et al. 2009). Fungi are not able to penetrate the outer rigid bark of trees but are inoculated into the phloem tissue after successful host colonisation by bark beetles (Franceschi et al. 2000). Once inside the phloem, fungi trigger extensive hypersensitive reactions around the infection site with very high accumulation of specialised metabolites resulting in necrosis of phloem tissues (Berryman 1972; Erbilgin et al. 2006; Franceschi et al. 2005; Lieutier et al. 2009; Zhao et al. 2011a, b). Furthermore, fungi can deplete or consume tree reserves such as NSC and lipids that are necessary to synthesise new inducible defence chemicals against invaders (Lahr and Krokene 2013). In this way, fungi might benefit from the stored tree resources to establish itself in the tree and consequently, reduce the ability of the tree (exhaust tree defences) to mount new resistance against pathogens. After successfully colonising phloem and sapwood, fungi appear to remobilise the nutrients back to the phloem for the benefit of larvae and callow adults. Nutritional hypothesis putforth by Six and Elser (2020) and Six and Wingfield (2011) suggests that fungi provide nutrition for both primary and secondary bark beetles. Symbiotic fungi concentrate nitrogen and phosphorous close to beetle’s feeding galleries and can fulfil the nutritional requirement of larvae and callow adults (Six 2019; Six and Elser 2020). Moreover, they supply essential dietary nutrients such as vitamins, amino acids and also ergosterol, a fungal sterol essential for oogenesis and larval development that are absent or only present at low concentrations in the phloem (Bentz and Six 2006; Davis et al. 2019). However, provision of nutrients by fungi for their beetle vectors first requires exhaustion of tree defences.

### Role of fungi in modulating beetle colonisation

Increasing evidence suggests that fungal symbionts of bark beetles, in addition to overwhelming tree defence and improving beetle nutrition, have multiple roles in the life cycles of these insects. For example, ophiostomatoid fungi could serve as a source of bark beetle semiochemicals (Fig. 1 and Table 2) and may influence the behaviour of both conspecifics and heterospecifics (Kandasamy et al. 2016). The anti-aggregation or spacing pheromone verbenone produced by symbiotic microbes plays a major role in minimising intraspecific competition among offspring for the resource that directly affects their performance (Bakke 1981; Byers 1989). By inhibiting newly arriving beetles, fungi also benefit from avoiding direct competition for resources from new microbes introduced by conspecifics or heterospecifics (Cale et al. 2019). Interestingly, recent studies have shown that fungal symbionts of *I. typographus* such as *G. europhioides* and *G. penicillata* produce aggregation pheromone components of bark beetles (Table 2). Beetle pheromonal compounds such as 2-methyl-3-buten-2-ol, *exo*- and *endo*-brevicomin, and *trans*-conophthorin are synthesised by fungi de novo. This leads to an intriguing hypothesis that both beetles and their symbiotic fungi could have convergently evolved chemical communication signals that mutually benefit each other (Zhao et al. 2015, 2019a). However, ecological relevance for fungi that synthesise and release these bark beetle pheromones in nature is yet to be investigated. Fungal symbionts of *I. typographus* also produce a mixture of simple aliphatic, aromatic alcohols and their acetate esters. These compounds may help callow adult beetles during their maturation phase to feed on symbionts with high nutritional quality and actively select and disperse with beneficial fungi to a new host tree (Kandasamy et al. 2019, 2016).

### Responses of associated fungi to specialised metabolites

In addition to provision of nutrients, fungi alter host tissues by metabolising host tree defence compounds such as terpenoids and phenolics, which are toxic to both bark beetles and fungi (Krokene 2015; Raffa et al. 2015). As discussed in Sect. 3.4, phenolics have shown both anti-feedant and anti-nutritional effects on *I. typographus* in bioassays (Faccoli and Schlyter 2007; Hammerbacher et al. 2019); yet seem to have little effect on ophiostomatoid fungi. Interestingly, some fungi are able to degrade phenolics and utilise them as a carbon source (Wadke et al. 2016). Fungal species appear to differ in their ability to metabolise phenolics and their efficiency is positively correlated with fungal virulence (Zhao et al. 2019b). Different fungi within the symbiotic community of *I. typographus* can fulfil the role of metabolising host tree defences, thus supporting bark beetles in successful colonisation and brood production (Zhao et al. 2019b). In multi-choice (cafeteria) bioassays, beetles did not discriminate strictly among fungi based on their degradation efficiency; yet, different virulent fungi that effectively degrade phenolics such as *E. polonica*, *G. penicillata* and *G. europhioides* were equally preferred by tunnelling adult beetles over other less virulent fungi (Kandasamy et al. 2019; Zhao et al. 2019b). Moreover, the beetle’s preference towards virulent fungi is mediated by olfactory cues. The volatile profile of *E. polonica*, *G. penicillata* and *G. europhioides* was highly attractive to the young adults of *I. typographus*, whereas volatiles from avirulent *Ophiostoma* species were unattractive (Kandasamy et al. 2019). These results collectively show that the fungal community of *I. typographus* forms a redundant and interchangeable group, and the loss of one fungus could be compensated by another fungal species performing similar functions such as detoxification of phenolics.

### Effect of temperature and drought on fungus-tree interactions

The growth of ophiostomatoid fungi is influenced by various factors such as phloem temperature, moisture and chemistry (Six 2012). Based on their temperature optima, the fungal community of *I. typographus* can be classified into two groups: cold-tolerant fungi including *E. polonica*, *G. penicillata* and *G. europhioides* with a temperature optimum of around 20–23 °C, and warm tolerant fungi such as *O. bicolor* with optimum temperatures of around 30 °C (Solheim 1991). These variations in temperature optima explain regional differences in the composition of fungal communities with *O. bicolor* dominating in warmer regions over *E. polonica* and *Grosmannia* species being prevalent in colder regions (Marin et al. 2005; Solheim 1991).

Increasing temperatures are often accompanied by drought, predisposing trees to bark beetle and fungal attack. Drought, via the altered provision of host defence chemicals, presumably influences the formation of the hypersensitive wound reaction zones in response to pathogens (Netherer et al. 2016). Longer lesions indicate that trees are susceptible to pathogen attack or that the pathogen is highly virulent, whereas shorter lesions indicate that trees are resistant to pathogen attack or that the pathogen is less virulent (Christiansen et al. 1987). Drought alone does not drastically change the constitutive chemical defences of trees in the short term but reduces the hydrolytic conductivity of the vascular tissues (Croisé et al. 2001). However, drought shows clear effects on a tree’s chemical response when challenged with pathogens or herbivores (Klutsch et al. 2017). Experiments conducted with conifer seedlings and field grown trees with modified water availability showed that tree age plays an important role in resistance or susceptibility to a particular pathogen. Norway spruce trees, subject to mild drought for several months, increased resistance (short lesion) to the pathogen *E. polonica* when inoculated experimentally (Christiansen and Glosli 1996). Similarly, mild drought stress on hybrid pine (*P. contorta* x *P. banksiana*) trees resulted in shorter lesion lengths after inoculation with *G. clavigera* compared to well-watered trees (Arango-Velez et al. 2014). In contrast, Norway spruce seedlings exposed to mild drought conditions were susceptible to pathogens (long lesions and high mortality of seedlings) compared to healthy seedlings (Linnakoski et al. 2017). Similarly, mild water stress on *Pinus taeda* seedlings resulted in higher pathogenicity of *Leptographium terebrantis* compared to normal water conditions (Devkota et al. 2018). Additionally, susceptibility of conifers to pathogens under different levels of drought stress is determined by the type of fungal species and is also strain specific (Devkota et al. 2018; Linnakoski et al. 2017). These findings suggest that conifer seedlings are susceptible to pathogen attack under mild drought stress. On the other hand, mild drought could enhance the resistance of mature trees to pathogen attack with potential negative implications for beetle colonisation and reproduction.

## Concluding remarks and outlook on future research directions

### Effects of drought on bark beetle-induced Norway spruce mortality are still poorly understood

Despite intense research on Norway spruce defence mechanisms, *I. typographus* population dynamics and the role of fungal symbionts in beetle life history, knowledge on the particular effects of drought on these multitrophic relationships is still surprisingly scarce. While severe precipitation deficits have been recognised to increase the risk of bark beetle outbreaks decades ago (Merker 1956; Worrell 1983; Zinecker 1957), we still do not fully understand the physiological and biochemical processes explaining drought-mediated *I. typographus* attack. In a changing climate, various abiotic stress factors such as increasing temperatures and rising levels of atmospheric CO_2_ potentially interact in synergistic or antagonistic ways and affect tree resistance to biotic stressors (Holopainen et al. 2018; Niinemets 2010). Thus, field observations gained after natural drought events may strongly differ from experimental data obtained from (semi-) controlled conditions (Allen et al. 2010; Berini et al. 2018). Controlled greenhouse studies with clone seedlings, saplings, or even cell cultures (Messner and Schröder 1999) can reduce the interference of external factors, whereas field experiments using bioassays on mature forest trees (Gaylord et al. 2013; Netherer et al. 2015; Turcani and Nakladal 2007) simulate more natural but highly fluctuating conditions. Moreover, relationships among tree substrate, beetle and fungi are complex and only tree-beetle or beetle-fungus interactions have been studied under laboratory or in field conditions (Hammerbacher et al. 2019; Kandasamy et al. 2019; Linnakoski et al. 2017; Solheim 1991; Wadke et al. 2016; Zhao et al. 2019b). While moderate drought seems to overall strengthen tree defences, multiple stressors can increase tree susceptibility to pathogens and bark beetles. To fully unravel the effects of drought on interactions among trees, fungi, and bark beetles, multifaceted experimental approaches in field and laboratory conditions including molecular biology (Chakraborty et al. 2020a, b; Powell et al. 2020) should focus on multiple interacting partners in the bark beetle system.

### Future research directions

Future work should focus on all the players in the multipartite relationship between Norway spruce, *I. typographus*, and associated microbes and combine chemo-ecological, molecular and behavioural approaches.

1. Comprehensively evaluate **Norway spruce** stress and defence status, involving stress history and molecular mechanisms underlying stress-related biochemical processes by:
2. Tracing the allocation, storage and mobilisation of NSC within the tree using CO_2_ isotope labelling and measuring multiple tree parameters to better understand carbon source and sink dynamics and to unravel trade-offs between primary and secondary metabolism of Norway spruce, as recently proposed by Huang et al. (2019) but with specific focus on drought effects.
3. Linking molecular, biochemical and physiological markers of tree drought stress such as capacity of specialised metabolite biosynthesis and indicators of tree water potential to bark beetle attack and colonisation success in order to improve assessment of stress-related Norway spruce susceptibility to *I. typographus* infestation.
4. Provide an updated, comprehensive view on ***I. typographus-***Norway spruce interactions considering behavioural sequence from dispersal to acceptance of host tissue by:
5. Studying attraction, host selection and acceptance from landscape level to bark sections of individual trees in (semi-) field and laboratory bioassays, to clearly identify the decisive environmental and tree parameters for host choice.
6. Relating visual, olfactory, gustatory and haptic cues from the forest environment to the basic sensory mechanisms of host identification and selection to allow deeper insights in the molecular biology and ecology of *I. typographus*, using the recently available genome (Powell et al. 2020).
7. Taking advantage of dropping costs of modern DNA sequencing methods, transcriptomic, proteomic and even whole-genome data for *I. typographus* should be generated under various biotic and abiotic interactions. Such analyses support a deeper understanding of underlying mechanisms of *I. typographus* life history and interactions with its environment and provide an opportunity for large-scale comparative studies to understand the evolution of bark beetles and other herbivorous insects.
8. Improve our understanding of the role of **fungal symbionts** on *I. typographus* performance by:
9. Elucidating nutritional and detoxification roles of fungal symbionts by assessing the performance or fitness of bark beetles in the presence and absence of fungal symbionts in terpenes or phenolics-enriched diet. Tree chemicals should be tested as single compounds and as mixtures to better understand synergistic properties of diverse spruce defence chemicals on bark beetles and fungi.
10. Focusing on how drought affects the growth rates of ophiostomatoid fungi in phloem and consequent effects on their production of volatile cues that mediate interactions between fungi and beetles to better understand multitrophic interactions under changing climatic conditions.
11. Field trapping of *I. typographus* using live cultures of fungi or synthetic olfactory-active fungal volatiles to improve our knowledge on the role of fungi in host choice, aggregation and anti-aggregation of *I. typographus.*
12. Quantifying the relationship between fungal establishment in the phloem and insect semiochemical production to elucidate the role of fungal volatiles in successful tree attack and brood establishment.

### Conclusions

Tree resistance, insect and pathogen performance are strongly modified by the intensity, timing and duration of drought events. A clear definition of ‘moderate’ versus 'severe’ and ‘acute’ as opposed to 'chronic’ water deficit is necessary for a meaningful interpretation of multiple studies on drought-mediated effects (Caldeira 2019; Gely et al. 2020). A known fact is that trees’ resilience to adapt to constant, persistent unfavourable site conditions can enhance their potential to endure drought periods and also to defend biotic invaders (Berini et al. 2018; Ferrenberg et al. 2017). These chronically stressed trees seem to be less susceptible to *I. typographus* infestations than trees suddenly exposed to drought as well as fast growing trees of high tissue quality but moderately impaired defence (Baier et al. 2002; Blomqvist et al. 2018; Netherer et al. 2019).

A focus on surviving trees, i.e. trees without attacks or those which succeeded to fight-off invasions even at high attack pressure (Balogh et al. 2018; Schiebe et al. 2012; Six et al. 2018), can help to reveal the causalities of *I. typographus* infestations. In natural forests, bark beetle outbreaks likely act as natural selection events in favour of tree genotypes better adapted to warm and dry conditions and escaping *I. typographus* attacks (Jakuš et al. 2011). The future mitigation of drought-related forest mortality also relies on the promotion of particular tree species and genotypes with enhanced individual defence capacity (Telford et al. 2014). High species diversity and natural species communities are expected to stimulate selective processes that enhance the resistance of European forests to specialised herbivores and pathogens (Grossiord 2019) such as *I. typographus* and its associated organisms.

## References

[CR1] Adams HD, Germino MJ, Breshears DD, Barron-Gafford GA, Guardiola-Claramonte M, Zou CB, Huxman TE (2013). Nonstructural leaf carbohydrate dynamics of *Pinus edulis* during drought-induced tree mortality reveal role for carbon metabolism in mortality mechanism. New Phytol.

[CR2] Allen CD, Macalady AK, Chenchouni H (2010). A global overview of drought and heat-induced tree mortality reveals emerging climate change risks for forests. For Ecol Manag.

[CR3] Andersson MN (2012). Mechanisms of odor coding in coniferous bark beetles: From neuron to behavior and application. Psyche (Camb Mass).

[CR4] Andersson MN, Larsson MC, Schlyter F (2009). Specificity and redundancy in the olfactory system of the bark beetle *Ips typographus*: single-cell responses to ecologically relevant odors. J Insect Physiol.

[CR5] Andersson MN, Larsson MC, Blazenec M, Jakuš R, Zhang QH, Schlyter F (2010). Peripheral modulation of pheromone response by inhibitory host compound in a beetle. J Exp Biol.

[CR6] Andersson MN, Grosse-Wilde E, Keeling C (2013). Antennal transcriptome analysis of the chemosensory gene families in the tree killing bark beetles, *Ips typographus* and *Dendroctonus ponderosae* (Coleoptera: Curculionidae: Scolytinae). BMC Genom.

[CR7] Andersson MN, Löfstedt C, Newcomb RD (2015). Insect olfaction and the evolution of receptor tuning. Front Ecol Evol.

[CR8] Arango-Velez A, Gonzalez LM, Meents MJ (2014). Influence of water deficit on the molecular responses of *Pinus contorta* x *Pinus banksiana* mature trees to infection by the mountain pine beetle fungal associate, *Grosmannia clavigera*. Tree Physiol.

[CR9] Ayres MP, Lombardero MJ (2000). Assessing the consequences of global change for forest disturbance from herbivores and pathogens. Sci Total Environ.

[CR10] Baier P, Führer E, Kirisits T, Rosner S (2002). Defence reactions of Norway spruce against bark beetles and the associated fungus *Ceratocystis polonica* in secondary pure and mixed species stands. Forest Ecol Manag.

[CR11] Bakke A (1976). Spruce bark beetle, *Ips typographus*: Pheromone production and field response to synthetic pheromones. Naturwissenschaften.

[CR12] Bakke A (1981). Inhibition of the response in *Ips typographus* to the aggregation pheromone; field evaluation of verbenone and ipsenol. Z Angew Entomol.

[CR13] Balogh SL, Huber DPW, Lindgren BS (2018). Single-generation effects on terpenoid defenses in lodgepole pine populations following mountain pine beetle infestation. PLoS One.

[CR14] Bansal S, Germino MJ (2009). Temporal variation of nonstructural carbohydrates in montane conifers: similarities and differences among developmental stages, species and environmental conditions. Tree Physiol.

[CR15] Bentz B, Six DL (2006). Ergosterol content of fungi associated with *Dendroctonus ponderosae* and *Dendroctonus rufipennis* (Coleoptera: Curculionidae, Scolytinae). Ann Entomol Soc Am.

[CR16] Bentz BJ, Jönsson AM, Schroeder M, Weed A, Wilcke RAI, Larsson K (2019). *Ips typographus* and *Dendroctonus ponderosae* models project thermal suitability for intra- and inter-continental establishment in a changing climate. Front For Glob.

[CR17] Berini JL, Brockman SA, Hegeman AD, Reich PB, Muthukrishnan R, Montgomery RA, Forester JD (2018). Combinations of abiotic factors differentially alter production of plant secondary metabolites in five woody plant species in the boreal-temperate transition zone. Front Plant Sci.

[CR18] Berryman AA (1972). Resistance of conifers to invasion by bark beetle-fungus associations. Bioscience.

[CR19] Biedermann PHW, Muller J, Grégoire J-C (2019). Bark beetle population dynamics in the anthropocene: challenges and solutions. Trends Ecol Evol.

[CR20] Binyameen M, Jankuvová J, Blaženec M (2014). Co-localization of insect olfactory sensory cells improves the discrimination of closely separated odour sources. Funct Ecol.

[CR21] Birgersson G, Bergström G (1989). Volatiles released from individual spruce bark beetle entrance holes: quantitative variations during the first week of attack. J Chem Ecol.

[CR22] Birgersson G, Schlyter F, Löfqvist J, Bergström G (1984). Quantitative variation of pheromone components in the spruce bark beetle *Ips typographus* from different attack phases. J Chem Ecol.

[CR23] Blažytė-Čereškienė L, Apšegaitė V, Radžiutė S, Mozūraitis R, Būda V, Pečiulytė D (2015). Electrophysiological and behavioural responses of *Ips typographus* (L.) to trans-4-thujanol—a host tree volatile compound. Ann For Sci.

[CR24] Blomquist GJ, Figueroa-Teran R, Aw M (2010). Pheromone production in bark beetles. Insect Biochem Mol Biol.

[CR25] Blomqvist M, Kosunen M, Starr M, Kantola T, Holopainen M, Lyytikäinen-Saarenmaa P (2018). Modelling the predisposition of Norway spruce to *Ips typographus* L. infestation by means of environmental factors in southern Finland. Eur J For Res.

[CR26] Borg-Karlson AK, Lindström M, Norin T, Persson M, Valterová I (1993). Enantiomeric composition of monoterpene hydrocarbons in different tissues of Norway spruce, *Picea abies* (L) Karst. A multi-dimensional gas chromatography study. Acta Chem Scand.

[CR27] Branco M, Pereira JS, Mateus E, Tavares C, Paiva MR (2010). Water stress affects *Tomicus destruens* host pine preference and performance during the shoot feeding phase. Ann For Sci.

[CR28] Brignolas F, Lacroix B, Lieutier F (1995). lnduced responses in phenolic metabolism in two Norway spruce clones after wounding and inoculations with *Ophiostoma polonicum*, a bark beetle-associated fungus. Plant Physiol.

[CR29] Brignolas F, Lieutier F, Sauvard D, Christiansen E, Berryman AA (1998). Phenolic predictors for Norway spruce resistance to the bark beetle *Ips typographus* (Coleoptera: Scolytidae) and an associated fungus, *Ceratocystis polonica*. Can J For Res.

[CR30] Bruce TJA, Pickett JA (2011). Perception of plant volatile blends by herbivorous insects – Finding the right mix. Phytochemistry.

[CR31] Bryant JP, Chapin FS, Klein DR (1983). Carbon/nutrient balance of boreal plants in relation to vertebrate herbivory. Oikos.

[CR32] Byers JA (1989). Chemical ecology of bark beetles. Experientia.

[CR33] Byers JA (1999). Effects of attraction radius and flight paths on catch of scolytid beetles dispersing outward through rings of pheromone traps. J Chem Ecol.

[CR34] Byers JA (2000). Wind-aided dispersal of simulated bark beetles flying through forests. Ecol Model.

[CR35] Byers JA, Birgersson G (1990). Pheromone production in a bark beetle independent of myrcene precursor in host pine species. Naturwissenschaften.

[CR36] Byers JA, Zhang Q-H, Schlyter F, Birgersson G (1998). Volatiles from nonhost birch trees inhibit pheromone response in spruce bark beetles. Naturwissenschaften.

[CR37] Byers JA, Zhang QH, Birgersson G (2000). Strategies of a bark beetle, *Pityogenes bidentatus*, in an olfactory landscape. Naturwissenschaften.

[CR38] Caldeira MC (2019). The timing of drought coupled with pathogens may boost tree mortality. Tree Physiol.

[CR39] Cale JA, Ding R, Wang F, Rajabzadeh R, Erbilgin N (2019). Ophiostomatoid fungi can emit the bark beetle pheromone verbenone and other semiochemicals in media amended with various pine chemicals and beetle-released compounds. Fungal Ecol.

[CR40] Celedon JM, Bohlmann J (2019). Oleoresin defenses in conifers: chemical diversity, terpene synthases and limitations of oleoresin defense under climate change. New Phytol.

[CR41] Chakraborty A, Ashraf MZ, Modlinger R, Synek J, Schlyter F, Roy A (2020). Unravelling the gut bacteriome of Ips (Coleoptera: Curculionidae: Scolytinae): identifying core bacterial assemblage and their ecological relevance. Sci Rep.

[CR42] Chakraborty A, Modlinger R, Ashraf MZ, Synek J, Schlyter F, Roy A (2020). Core mycobiome and their ecological relevance in the gut of five Ips bark beetles (Coleoptera: Curculionidae: Scolytinae). Front Microbiol.

[CR43] Chang R, Duong TA, Taerum SJ, Wingfield MJ, Zhou X, Yin M, de Beer ZW (2019). Ophiostomatoid fungi associated with the spruce bark beetle *Ips typographus*, including 11 new species from China. Persoonia.

[CR44] Chong J, Poutaraud A, Hugueney P (2009). Metabolism and roles of stilbenes in plants. Plant Sci.

[CR45] Christiansen E, Glosli AM (1996) Mild drought enhances the resistance of Norway spruce to a bark beetle-transmitted blue-stain fungus. vol NC-183. USDA Forest Service Gen. Tech. Rep., St. Paul, MN 55108

[CR46] Christiansen E, Waring RH, Berryman AA (1987). Resistance of conifers to bark beetle attack: searching for general relationships. For Ecol Manag.

[CR47] Coulson RN, Pulley PE, Pope DN, Fargo WS, Gagne JA, Kelly CL (1980) Estimation of survival and allocation of adult southern pine beetles between trees during the development of an infestation. In: Berryman AA, Safranyik L (eds) Proceedings of the second IUFRO conference on dispersal of forest insects: evaluation, theory and management implications. Washington State University, Pullman, USA, Sandpoint, Idaho, USA, pp 194–212

[CR48] Croisé L, Lieutier F, Cochard H, Dreyer E (2001). Effects of drought stress and high density stem inoculations with *Leptographium wingfieldii* on hydraulic properties of young Scots pine trees. Tree Physiol.

[CR49] Davis TS (2015). The ecology of yeasts in the bark beetle holobiont: a century of research revisited. Microb Ecol.

[CR50] Davis TS, Stewart JE, Mann A, Bradley C, Hofstetter R (2019). Evidence for multiple ecological roles of *Leptographium abietinum*, a symbiotic fungus associated with the North American spruce beetle. Fungal Ecol.

[CR51] Devkota P, Enebak SA, Eckhardt LG (2018). The impact of drought and vascular-inhabiting pathogen invasion in *Pinus taeda* health. Int J For Res.

[CR52] Dickens JC (1981). Behavioural and electro-physiological responses of the bark beetle *Ips typographus* to potential pheromone components. Physiol Entomol.

[CR53] Eldhuset TD, Nagy NE, Volařík D, Børja I, Gebauer R, Yakovlev IA, Krokene P (2013). Drought affects tracheid structure, dehydrin expression, and above- and belowground growth in 5-year-old Norway spruce. Plant Soil.

[CR54] Elkinton JS, Wood DL (1980). Feeding and boring behavior of the bark beetle *Ips paraconfusus* (Coleoptera: Scolytidae) on the bark of a host and non-host tree species. Can Entomol.

[CR55] Erbilgin N, Krokene P, Christiansen E, Zeneli G, Gershenzon J (2006). Exogenous application of methyl jasmonate elicits defenses in Norway spruce (*Picea abies*) and reduces host colonization by the bark beetle *Ips typographus*. Oecologia.

[CR56] Erbilgin N, Krokene P, Kvamme T, Christiansen E (2007). A host monoterpene influences *Ips typographus* (Coleoptera: Curculionidae, Scolytinae) responses to its aggregation pheromone. Agr For Entomol.

[CR57] Erbilgin N, Cale JA, Hussain A, Ishangulyyeva G, Klutsch JG, Najar A, Zhao S (2017). Weathering the storm: how lodgepole pine trees survive mountain pine beetle outbreaks. Oecologia.

[CR58] Evensen PC, Solheim H, Høiland K, Stenersen J (2000). Induced resistance of Norway spruce, variation of phenolic compounds and their effects on fungal pathogens. For Pathol.

[CR59] Everaerts C, Grégoire JC, Merlin J, Mattson WJ, Levieux J, Bernard-Dagan C (1988). The toxicity of Norway spruce monoterpenes to two bark beetle species and their associates. Mechanisms of woody plant defences against insects. Search for pattern.

[CR60] Eyles A, Bonello P, Ganley R, Mohammed C (2010). Induced resistance to pests and pathogens in trees. New Phytol.

[CR61] Faccoli M, Schlyter F (2007). Conifer phenolic resistance markers are bark beetle antifeedant semiochemicals. Agr For Entomol.

[CR62] Faccoli M, Blazenec M, Schlyter F (2005). Feeding response to host and nonhost compounds by males and females of the spruce bark beetle *Ips typographus* in a tunneling microassay. J Chem Ecol.

[CR63] Felicijan M, Novak M, Kraševec N, Urbanek Krajnc A (2015). Antioxidant defences of Norway spruce bark against bark beetles and its associated blue-stain fungus. Agricultura.

[CR64] Felicijan M, Kristl J, Krajnc AU (2016). Pre-treatment with salicylic acid induces phenolic responses of Norway spruce (*Picea abies*) bark to bark beetle (I*ps typographus*) attack. Trees.

[CR65] Ferrenberg S, Kane JM, Langenhan JM (2015). To grow or defend? Pine seedlings grow less but induce more defences when a key resource is limited. Tree Physiol.

[CR66] Ferrenberg S, Langenhan JM, Loskot SA, Rozal LM, Mitton JB (2017). Resin monoterpene defenses decline within three widespread species of pine (*Pinus*) along a 1530-m elevational gradient. Ecosphere.

[CR67] Fossdal CG, Nagy NE, Johnsen O, Dalen LS (2007). Local and systemic stress responses in Norway spruce: similarities in gene expression between a compatible pathogen interaction and drought stress. Physiol Mol Plant P.

[CR68] Franceschi VR, Krokene P, Krekling T, Christiansen E (2000). Phloem parenchyma cells are involved in local and distant defense responses to fungal inoculation or bark-beetle attack in Norway spruce (Pinaceae). Am J Bot.

[CR69] Franceschi VR, Krokene P, Christiansen E, Krekling T (2005). Anatomical and chemical defenses of conifer bark against bark beetles and other pests. New Phytol.

[CR70] Francke W, Vité JP (1983). Oxygenated terpenes in pheromone systems of bark beetles. J Appl Entomol.

[CR71] Franklin AJ, Debruyne C, Grégoire J-C (2000). Recapture of *Ips typographus* L. (Col., Scolytidae) with attractants of low release rates: localized dispersion and environmental influences. Agr For Entomol.

[CR72] Gaylord ML, Kolb TE, Pockman WT (2013). Drought predisposes pinon-juniper woodlands to insect attacks and mortality. New Phytol.

[CR73] Gely C, Laurance SGW, Stork NE (2020). How do herbivorous insects respond to drought stress in trees?. Biol Rev Camb Philos Soc.

[CR74] Ghimire RP, Kivimäenpää M, Blomqvist M, Holopainen T, Lyytikäinen-Saarenmaa P, Holopainen JK (2016). Effect of bark beetle (*Ips typographus* L.) attack on bark VOC emissions of Norway spruce (*Picea abies* Karst.) trees. Atmos Environ.

[CR75] Graham K (1959). Release by flight exercise of a chemotropic response from photopositive domination in a scolytid beetle. Nature.

[CR76] Grossiord C (2019). Having the right neighbors: how tree species diversity modulates drought impacts on forests. New Phytol.

[CR77] Hallberg E (1982). Sensory organs in *Ips typographus* (Insecta: Coleoptera)—fine structure of the sensilla of the maxillary and labial palps. Acta Zool.

[CR78] Hammerbacher A, Ralph SG, Bohlmann J, Fenning TM, Gershenzon J, Schmidt A (2011). Biosynthesis of the major tetrahydroxystilbenes in spruce, astringin and isorhapontin, proceeds via resveratrol and is enhanced by fungal infection. Plant Physiol.

[CR79] Hammerbacher A, Schmidt A, Wadke N (2013). A common fungal associate of the spruce bark beetle metabolizes the stilbene defenses of Norway spruce. Plant Physiol.

[CR80] Hammerbacher A, Paetz C, Wright LP (2014). Flavan-3-ols in Norway spruce: biosynthesis, accumulation, and function in response to attack by the bark beetle-associated fungus *Ceratocystis polonica*. Plant Physiol.

[CR81] Hammerbacher A, Raguschke B, Wright LP, Gershenzon J (2018). Gallocatechin biosynthesis via a flavonoid 3',5'-hydroxylase is a defense response in Norway spruce against infection by the bark beetle-associated sap-staining fungus *Endoconidiophora polonica*. Phytochemistry.

[CR82] Hammerbacher A, Kandasamy D, Ullah C, Schmidt A, Wright LP, Gershenzon J (2019). Flavanone-3-Hydroxylase plays an important role in the biosynthesis of spruce phenolic defenses against bark beetles and their fungal associates. Front Plant Sci.

[CR83] Hansson BS, Anton S (2000). Function and morphology of the antennal lobe: new developments. Annu Rev Entomol.

[CR84] Hansson BS, Stensmyr MC (2011). Evolution of insect olfaction. Neuron.

[CR85] Hartmann H, Moura CF, Anderegg WRL (2018). Research frontiers for improving our understanding of drought induced tree and forest mortality. New Phytol.

[CR86] Herms DA, Mattson WJ (1992). The dilemma of plants: to grow or defend. Q Rev Biol.

[CR87] Hietz P, Baier P (2005). Tree temperatures, volatile organic emissions, and primary attraction of bark beetles. Phyton Ann Rei Bot A.

[CR88] Hoch G, Richter A, Körner C (2003). Non-structural carbon compounds in temperate forest trees. Plant Cell Environ.

[CR89] Holopainen JK, Virjamo V, Ghimire RP, Blande JD, Julkunen-Tiitto R, Kivimaenpaa M (2018). Climate change effects on secondary compounds of forest trees in the Northern hemisphere. Front Plant Sci.

[CR90] Huang J, Hammerbacher A, Weinhold A (2019). Eyes on the future - evidence for trade-offs between growth, storage and defense in Norway spruce. New Phytol.

[CR91] Huang J, Kautz M, Trowbridge AM (2020). Tree defence and bark beetles in a drying world: carbon partitioning, functioning and modelling. New Phytol.

[CR92] Hussain A, Classens G, Guevara-Rozo S, Cale JA, Rajabzadeh R, Peters BR, Erbilgin N (2020). Spatial variation in soil available water holding capacity alters carbon mobilization and allocation to chemical defenses along jack pine stems. Environ Exp Bot.

[CR93] Ishangulyyeva G, Najar A, Curtis JM, Erbilgin N (2016). Fatty acid composition of novel host jack pine do not prevent host acceptance and colonization by the invasive mountain pine beetle and its symbiotic fungus. PLoS One.

[CR94] Jackson GE, Irvine J, Grace J, Khalil AAM (1995). Abscisic acid concentrations and fluxes in droughted conifer saplings. Plant Cell Environ.

[CR95] Jacquet JS, Bosc A, O'Grady A, Jactel H (2014). Combined effects of defoliation and water stress on pine growth and non-structural carbohydrates. Tree Physiol.

[CR96] Jactel H, Petit J, Desprez-Loustau M-L, Delzon S, Piou D, Battisti A, Koricheva J (2012). Drought effects on damage by forest insects and pathogens: a meta-analysis. Glob Change Biol.

[CR97] Jakoby O, Lischke H, Wermelinger B (2019). Climate change alters elevational phenology patterns of the European spruce bark beetle (*Ips typographus*). Glob Change Biol.

[CR98] Jakuš R, Edwards-Jonášová M, Cudlín P, Blaženec M, Ježík M, Havlíček F, Moravec I (2011). Characteristics of Norway spruce trees (*Picea abies*) surviving a spruce bark beetle (*Ips typographus L*.) outbreak. Trees.

[CR99] Kainulainen PJ, Oksanen J, Palomäki V, Holopainen JK, Holopainen T (1992). Effect of drought and waterlogging stress on needle monoterpenes of *Picea abies*. Can J Bot.

[CR100] Kalinová B, Brizova R, Knizek M, Turcani M, Hoskovec M (2014). Volatiles from spruce trap-trees detected by *Ips typographus* bark beetles: chemical and electrophysiological analyses. Arthropod Plant Interact.

[CR101] Kandasamy D, Gershenzon J, Hammerbacher A (2016). Volatile organic compounds emitted by fungal associates of conifer bark beetles and their potential in bark beetle control. J Chem Ecol.

[CR102] Kandasamy D, Gershenzon J, Andersson MN, Hammerbacher A (2019). Volatile organic compounds influence the interaction of the Eurasian spruce bark beetle (*Ips typographus*) with its fungal symbionts. ISME J.

[CR103] Kännaste A, Zhao T, Lindström A, Stattin E, Långström B, Borg-Karlson A-K (2012). Odors of Norway spruce (*Picea abies* L.) seedlings: differences due to age and chemotype. Trees.

[CR104] Kausrud KL, Grégoire J-C, Skarpaas O, Erbilgin N, Gilbert M, Økland B, Stenseth NC (2011). Trees Wanted—Dead or Alive! Host selection and population dynamics in tree-killing bark beetles. PLoS One.

[CR105] Kausrud K, Økland B, Skarpaas O, Grégoire J-C, Erbilgin N, Stenseth NC (2012). Population dynamics in changing environments: the case of an eruptive forest pest species. Bio Rev.

[CR106] Kelsey RG, Gallego D, Sánchez-García FJ, Pajares JA (2014). Ethanol accumulation during severe drought may signal tree vulnerability to detection and attack by bark beetles. Can J For Res.

[CR107] Kirisits T, Lieutier F, Day KR, Battisti A, Grégoire J-C, Evans HF (2004). Fungal associates of European bark beetles with special emphasis on the ophiostomatoid fungi. Bark and wood boring insects in living trees in Europe, a synthesis.

[CR108] Klutsch JG, Shamoun SF, Erbilgin N (2017). Drought stress leads to systemic induced susceptibility to a nectrotrophic fungus associated with mountain pine beetle in *Pinus banksiana* seedlings. PLoS One.

[CR109] Kohnle U, Vité JP, Baader EJ, Meyer H, Francke W (1991). Chirality of ipsdienol and ipsenol indicates a frass pheromone system in the spruce engraver, *Ips typographus*. Naturwissenschaften.

[CR110] Kolb TE, Fettig CJ, Ayres MP (2016). Observed and anticipated impacts of drought on forest insects and diseases in the United States. For Ecol Manag.

[CR111] Kolb T, Keefover-Ring K, Burr SJ, Hofstetter R, Gaylord M, Raffa KF (2019). Drought-mediated changes in tree physiological processes weaken tree defenses to bark beetle attack. J Chem Ecol.

[CR112] Koricheva J, Larsson S, Haukioja E, Keinänen M (1998). Regulation of woody plant secondary metabolism by resource availability: hypothesis testing by means of meta-analysis. Oikos.

[CR113] Körner C (2003). Carbon limitation in trees. J Ecol.

[CR114] Krokene P, Vega FE, Hofstetter R (2015). Conifer defense and resistance to bark beetles. Bark beetles: biology and ecology of native and invasive species.

[CR115] Krokene P, Solheim H, Christiansen E (2001). Induction of disease resistance in Norway spruce (*Picea abies*) by necrotizing fungi. Plant Pathol.

[CR116] Lahr EC, Krokene P (2013). Conifer stored resources and resistance to a fungus associated with the spruce bark beetle *Ips typographus*. PLoS One.

[CR117] Leufvén A, Birgersson G (1987). Quantitative variation of different monoterpenes around galleries of *Ips typographus* (Coleoptera:Scolytidae) attacking Norway spruce. Can J Bot.

[CR118] Leufvén A, Bergström G, Falsen E (1984). Interconversion of verbenols and verbenone by identified yeasts isolated from the spruce bark beetle *Ips typographus*. J Chem Ecol.

[CR119] Leufvén A, Bergström G, Falsen E (1988). Oxygenated monoterpenes produced by yeasts, isolated from *Ips typographus* (Coleoptera: Scolytidae) and grown in phloem medium. J Chem Ecol.

[CR120] Lewinsohn E, Gijzen M, Muzika RM, Barton K, Croteau RB (1993). Oleoresinosis in Grand fir (*Abies grandis*) saplings and mature trees. Plant Physiol.

[CR121] Li SH, Nagy NE, Hammerbacher A, Krokene P, Niu XM, Gershenzon J, Schneider B (2012). Localization of phenolics in phloem parenchyma cells of Norway spruce (*Picea abies*). Chembiochem Eur J Chem Biol.

[CR122] Lieutier F, Lieutier F, Day KR, Battisti A, Grégoire J-C, Evans HF (2004). Host resistance to bark beetles and its variations. Bark and wood boring insects in living trees in Europe - A Synthesis.

[CR123] Lieutier F, Brignolas F, Sauvard D, Yart A, Galet C, Brunet M, Van de Sype H (2003). Intra- and inter-provenance variability in phloem phenols of *Picea abies* and relationship to a bark beetle-associated fungus. Tree Physiol.

[CR124] Lieutier F, Yart A, Salle A (2009). Stimulation of tree defenses by ophiostomatoid fungi can explain attack success of bark beetles on conifers. Ann For Sci.

[CR125] Lindelöw Å, Risberg B, Sjödin K (1992). Attraction during flight of scolytids and other bark-and wood-dwelling beetles to volatiles from fresh and stored spruce wood. Can J For Res.

[CR126] Lindgren BS, Miller DR (2019). Effect of verbenone on five species of bark beetles (Coleoptera:Scolytidae) in lodgepole pine forests. Environ Entomol.

[CR127] Lindgren BS, Raffa KF (2013). Evolution of tree killing in bark beetles (Coleoptera: Curculionidae): trade-offs between the maddening crowds and a sticky situation. Can Entomol.

[CR128] Lindström M, Norin T, Birgersson G, Schlyter F (1989). Variation of enantiomeric composition of alpha-pinene in Norway spruce, *Picea abies*, and its influence on production of verbenol isomers by *Ips typographus* in the field. J Chem Ecol.

[CR129] Linnakoski R, de Beer ZW, Niemela P, Wingfield MJ (2012). Associations of conifer-infesting bark beetles and fungi in Fennoscandia. Insects.

[CR130] Linnakoski R, Sugano J, Junttila S, Pulkkinen P, Asiegbu FO, Forbes KM (2017). Effects of water availability on a forestry pathosystem: fungal strain-specific variation in disease severity. Sci Rep.

[CR131] Lombardero MJ, Ayres MP, Lorio PL, Ruel JJ (2000). Environmental effects on constitutive and inducible resin defences of *Pinus taeda*. Ecol Lett.

[CR132] Lopez-Goldar X, Villari C, Bonello P, Borg-Karlson AK, Grivet D, Zas R, Sampedro L (2018). Inducibility of plant secondary metabolites in the stem predicts genetic variation in resistance against a key insect herbivore in maritime pine. Front Plant Sci.

[CR133] Lusebrink I, Erbilgin N, Evenden ML (2016). The effect of water limitation on volatile emission, tree defense response, and brood success of *Dendroctonus ponderosae* in two pine hosts, Lodgepole, and Jack pine. Front Ecol Evol.

[CR134] Mageroy MH, Wilkinson SW, Tengs T (2020). Molecular underpinnings of methyl jasmonate-induced resistance in Norway spruce. Plant Cell Environ.

[CR135] Månsson PE (2005) Host selection and antifeedants in *Hylobius abietis* pine weevils. Dissertation, Swedish University of Agricultural Sciences

[CR136] Marin M, Preisig O, Wingfield BD, Kirisits T, Yamaoka Y, Wingfield MJ (2005). Phenotypic and DNA sequence data comparisons reveal three discrete species in the *Ceratocystis polonica* species complex. Mycol Res.

[CR137] Marini L, Økland B, Jönsson AM (2017). Climate drivers of bark beetle outbreak dynamics in Norway spruce forests. Ecography.

[CR138] Martin D, Tholl D, Gershenzon J, Bohlmann J (2002). Methyl jasmonate induces traumatic resin ducts, terpenoid resin biosynthesis, and terpenoid accumulation in developing xylem of Norway Spruce stems. Plant Physiol.

[CR139] Martin DM, Gershenzon J, Bohlmann J (2003). Induction of volatile terpene biosynthesis and diurnal emission by methyl jasmonate in foliage of Norway Spruce. Plant Physiol.

[CR140] Mattson WJ, Haak RA (1987). The role of drought in outbreaks of plant-eating insects. Drought's physiological effects on plants can predict its influence on insect populations. Bioscience.

[CR141] McDowell NG (2011). Mechanisms linking drought, hydraulics, carbon metabolism, and vegetation mortality. Plant Physiol.

[CR142] McDowell N, Sevanto S (2010). The mechanisms of carbon starvation: how, when, or does it even occur at all?. New Phytol.

[CR143] McDowell N, Pockman WT, Allen CD (2008). Mechanisms of plant survival and mortality during drought: why do some plants survive while others succumb to drought?. New Phytol.

[CR144] Merker E (1956). Die Abhängigkeit des biologischen Gleichgewichts des Großen Fichtenborkenkäfers vom Wasserhaushalt des Waldes - Dependency of biological equilibrium of the European spruce bark beetle from forest water supply. Waldhygiene.

[CR145] Messner B, Schröder P (1999). Burst amplifying system in cell suspension cultures of spruce (*Picea abies* L. Karst.): Modulation of elicitor-induced release of hydrogen peroxide (oxidative burst) by ionophores and salicylic acid. J Appl Bot Food Qual.

[CR146] Metsämuuronen S, Sirén H (2019). Bioactive phenolic compounds, metabolism and properties: a review on valuable chemical compounds in Scots pine and Norway spruce. Phytochem Rev.

[CR147] Moran E, Lauder J, Musser C, Stathos A, Shu M (2017). The genetics of drought tolerance in conifers. New Phytol.

[CR148] Mustaparta H, Tømmerås BÅ, Baeckström P, Bakke JM, Ohloff G (1984). Ipsdienol-specific receptor cells in bark beetles: structure-activity relationships of various analogues and of deuterium-labelled ipsdienol. J Comp Physiol A.

[CR149] Nagy NE, Fossdal CG, Krokene P, Krekling T, Lönneborg A, Solheim H (2004). Induced responses to pathogen infection in Norway spruce phloem:changes in polyphenolic parenchyma cells, chalcone synthasetranscript levels and peroxidase activity. Tree Physiol.

[CR150] Netherer S, Matthews B, Katzensteiner K (2015). Do water-limiting conditions predispose Norway spruce to bark beetle attack?. New Phytol.

[CR151] Netherer S, Ehn M, Blackwell E, Kirisits T (2016). Defence reactions of mature Norway spruce (*Picea abies*) before and after inoculation of the blue-stain fungus *Endoconidiophora polonica* in a drought stress experiment. Cent Eur For.

[CR152] Netherer S, Panassiti B, Pennerstorfer J, Matthews B (2019). Acute drought is an important driver of bark beetle infestation in Austrian Norway spruce stands. Front For Glob Change.

[CR153] Niinemets Ü (2010). Responses of forest trees to single and multiple environmental stresses from seedlings to mature plants: past stress history, stress interactions, tolerance and acclimation. Forest Ecol Manag.

[CR154] Novak M, Krajnc AU, Lah L (2013). Low-density *Ceratocystis polonica* inoculation of Norway spruce (*Picea abies*) triggers accumulation of monoterpenes with antifungal properties. Eur J For Res.

[CR155] Ormeno E, Mevy JP, Vila B, Bousquet-Melou A, Greff S, Bonin G, Fernandez C (2007). Water deficit stress induces different monoterpene and sesquiterpene emission changes in Mediterranean species. Relationship between terpene emissions and plant water potential. Chemosphere.

[CR156] Pentzold S, Burse A, Boland W (2017). Contact chemosensation of phytochemicals by insect herbivores. Nat Prod Rep.

[CR157] Persson M, Sjödin K, Borg-Karlson AK, Norin T, Ekberg I (1996). Relative amounts and enantiomeric compositions of monoterpene hydrocarbons in xylem and needles of *Picea abies*. Phytochemistry.

[CR158] Petterson EM, Boland W (2003). Potential parasitoid attractants, volatile composition throughout a bark beetle attack. Chemoecology.

[CR159] Popa V, Déziel E, Lavallée R, Bauce E, Guertin C (2012). The complex symbiotic relationships of bark beetles with microorganisms: a potential practical approach for biological control in forestry. Pest Manag Sci.

[CR160] Powell D, Groβe-Wilde E, Krokene P (2020). A highly contiguous genome assembly of a major forest pest, the Eurasian spruce bark beetle *Ips typographus*. BioRxiv.

[CR161] Raffa KF, Grégoire JC, Lindgren BS, Vega FE, Hofstetter RW (2015). Natural history and ecology of bark beetles. Bark beetles Biology and ecology of native and invasive species.

[CR162] Raffa KF, Andersson MN, Schlyter F (2016). Host selection by bark beetles: playing the odds in a high-stake game. Adv Insect Physiol.

[CR163] Renwick JAA, Hughes PR, Krull IS (1976). Selective production of cis- and trans-verbenol from (–)-and (+)-α-pinene by a bark beetle. Science.

[CR164] Rouault G, Candau J-N, Lieutier F, Nageleisen L-M, Martin J-C, Warzée N (2006). Effects of drought and heat on forest insect populations in relation to the 2003 drought in Western Europe. Ann For Sci.

[CR165] Sambaraju KR, Carroll AL, Aukema BH (2019). Multiyear weather anomalies associated with range shifts by the mountain pine beetle preceding large epidemics. For Ecol Manag.

[CR166] Sancho-Knapik D, Sanz MA, Peguero-Pina JJ, Niinemets U, Gil-Pelegrín E (2017). Changes of secondary metabolites in *Pinus sylvestris* L needles under increasing soil water deficit. Ann For Sci.

[CR167] Santos AM, Vasconcelos T, Mateus E, Farrall MH, Gomes da Silva MD, Paiva MR, Branco M (2006). Characterization of the volatile fraction emitted by phloems of four pinus species by solid-phase microextraction and gas chromatography-mass spectrometry. J Chromatogr A.

[CR168] Schiebe C, Hammerbacher A, Birgersson G (2012). Inducibility of chemical defenses in Norway spruce bark is correlated with unsuccessful mass attacks by the spruce bark beetle. Oecologia.

[CR169] Schiebe C, Unelius CR, Ganji S, Binyameen M, Birgersson G, Schlyter F (2019). Styrene, (+)-trans-(1R,4S,5S)-4-thujanol and oxygenated monoterpenes related to host stress elicit strong electrophysiological responses in the bark beetle *Ips typographus*. J Chem Ecol.

[CR170] Schlyter F, Birgersson G, Hardie J, Minks AK (1999). Forest Beetles. Pheromones of non-lepidopteran insects associated with agricultural plants.

[CR171] Schlyter F, Löfqvist J (1986). Response of walking spruce bark beetles *Ips typographus* to pheromone produced in different attack phases. Entomol Exp Appl.

[CR172] Schlyter F, Birgersson G, Byers JA, Löfqvist J, Bergström G (1987). Field response of spruce bark beetle, *Ips typographus*, to aggregation pheromone candidates. J Chem Ecol.

[CR173] Schlyter F, Birgersson G, Leufvén A (1989). Inhibition of attraction to aggregation pheromone by verbenone and ipsenol. J Chem Ecol.

[CR174] Schönwitz R, Kloos M, Merk L, ZIegler H (1990). Patterns of monoterpenes stored in the needles of Picea abies (L) Karst from several locations in mountainous regions of southern Germany. Trees.

[CR175] Seidl R, Muller J, Hothorn T, Bassler C, Heurich M, Kautz M (2016). Small beetle, large-scale drivers: how regional and landscape factors affect outbreaks of the European spruce bark beetle. J Appl Ecol.

[CR176] Sevanto S, McDowell NG, Dickman LT, Pangle R, Pockman WT (2014). How do trees die? A test of the hydraulic failure and carbon starvation hypotheses. Plant Cell Environ.

[CR177] Sharma A, Shahzad B, Rehman A, Bhardwaj R, Landi M, Zheng B (2019). Response of phenylpropanoidpPathway and the role of polyphenols in plants under abiotic stress. Molecules.

[CR178] Silvestrini E, Michelozzi M, Skroppa T, Brancaleoni E, Ciccioli P (2004). Characterisation of different clones of *Picea abies* (L.) Karst using head-space sampling of cortical tissues combined with enantioselective capillary gas chromatography for the separation of chiral and non-chiral monoterpenes. J Chromatogr A.

[CR179] Six DL (2012). Ecological and evolutionary determinants of bark beetle-fungus symbioses. Insects.

[CR180] Six DL (2013). The bark beetle holobiont: why microbes matter. J Chem Ecol.

[CR181] Six DL (2019). A major symbiont shift supports a major niche shift in a clade of tree-killing bark beetles. Ecol Entomol.

[CR182] Six DL, Elser JJ (2020). Mutualism is not restricted to tree-killing bark beetles and fungi: the ecological stoichiometry of secondary bark beetles, fungi, and a scavenger. Ecol Entomol.

[CR183] Six DL, Wingfield BD (2011). The role of phytopathogenicity in bark beetle–fungus symbioses: a challenge to the classic paradigm. Annu Rev Entomol.

[CR184] Six DL, Vergobbi C, Cutter M (2018). Are survivors different? Genetic-based selection of trees by mountain pine beetle during a climate change-driven outbreak in a high-elevation pine forest. Front Plant Sci.

[CR185] Solheim H (1991). Oxygen deficiency and spruce resin inhibition of growth of blue-stain fungi associated with *Ips typographus*. Mycol Res.

[CR186] Stadelmann G, Bugmann H, Wermelinger B, Bigler C (2014). Spatial interactions between storm damage and subsequent infestations by the European spruce bark beetle. For Ecol Manag.

[CR187] Tan W, Blake TJ (2006). Drought tolerance, abscisic acid and electrolyte leakage in fast-and slow-growing black spruce (*Picea mariana*) progenies. Physiol Plant.

[CR188] Tausz M, Wonisch A, Peters J, Jiménez MS, Morales D, Grill D (2001). Short-term changes in free radical scavengers and chloroplast pigments in *Pinus canariensis* needles as affected by mild drought stress. J Plant Physiol.

[CR189] Tausz M, Sircelj H, Grill D (2004). The glutathione system as a stress marker in plant ecophysiology: is a stress-response concept valid?. J Exp Bot.

[CR190] Telford A, Cavers S, Ennos RA, Cottrell JE (2014). Can we protect forests by harnessing variation in resistance to pests and pathogens?. Forestry.

[CR191] Tittiger C, Blomquist GJ (2017). Pheromone biosynthesis in bark beetles. Curr Opin Insect Sci.

[CR192] Tømmerås BÅ (1985). Specialization of the olfactory receptor cells in the bark beetle *Ips typographus* and its predator *Thanasimus formicarius* to bark beetle pheromones and host tree volatiles. J Comp Physiol A.

[CR193] Tømmerås BÅ, Mustaparta H (1984). Enhanced attraction of *Ips typographus* by adding exo-brevicomin to pheromone traps. Naturwissenschaften.

[CR194] Turcani M, Nakladal O (2007). The results of manipulated experiments with inoculation of *Ips typographus* (L., 1758) to spruce trees under various levels of water stress. J For Sci.

[CR195] Turtola S, Manninen AM, Rikala R, Kainulainen PJ (2003). Drought stress alters the concentration of wood terpenoids in Scots pine and Norway spruce seedlings. J Chem Ecol.

[CR196] Urbanek Krajnc A, Kristl J, Ivancic A (2011). Application of salicylic acid induces antioxidant defense responses in the phloem of *Picea abies* and inhibits colonization by *Ips typographus*. For Ecol Manag.

[CR197] Urbanek Krajnc A, Novak M, Felicijan M, Kraševec N, Lešnik M, Zupanec N, Komel R (2014). Antioxidative response patterns of Norway spruce bark to low-density *Ceratocystis polonica* inoculation. Trees.

[CR198] Viiri H, Annila E, Kitunen V, Niemelä P (2001). Induced responses in stilbenes and terpenes in fertilized Norway spruce after inoculation with blue-stain fungus, *Ceratocystis polonica*. Trees.

[CR199] Wadke N, Kandasamy D, Vogel H (2016). The bark-beetle-associated fungus, *Endoconidiophora polonica*, utilizes the phenolic defense compounds of its host as a carbon source. Plant Physiol.

[CR200] Wallin KF, Raffa KF (2002). Prior encounters modulate subsequent choices in host acceptance behavior by the bark beetle *Ips pini*. Entomol Exp Appl.

[CR201] Wang Y, Lim L, Madilao L, Lah L, Bohlmann J, Breuil C (2014). Gene discovery for enzymes involved in limonene modification or utilization by the mountain pine beetle-associated pathogen *Grosmannia clavigera*. Appl Environ Microb.

[CR202] Wijerathna AN, Evenden ML (2019). Energy use by the mountain pine beetle (Coleoptera: Curculionidae: Scolytinae) for dispersal by flight. Physiol Entomol.

[CR203] Witzell J, Martín JA (2008). Phenolic metabolites in the resistance of northern forest trees to pathogens—past experiences and future prospects. Can J For Res.

[CR204] Wood DL (1972) Selection and colonization of ponderosa pines by bark beetles. In: van Emden HF (ed) Insect/plant Relationships. Royal Entomological Society Symposium No. 6. Blackwell Scientific Publications Oxford, England, pp 101–107

[CR205] Worrell R (1983). Damage by the spruce bark beetle in south Norway 1970–80: A survey and factors affecting its occurrence. Medd fra Norsk Inst Skogforsk Rep Nor For Res Inst.

[CR206] Yuvaraj JK, Roberts RE, Sonntag Y (2020). Putative ligand binding sites of two functionally characterized bark beetle odorant receptors. Biorxiv.

[CR207] Zeneli G, Krokene P, Christiansen E, Krekling T, Gershenzon J (2006). Methyl jasmonate treatment of mature Norway spruce (*Picea abies*) trees increases the accumulation of terpenoid resin components and protects against infection by *Ceratocystis polonica*, a bark beetle-associated fungus. Tree Physiol.

[CR208] Zhang Q-H, Schlyter F (2003). Redundancy, synergism, and active inhibitory range of non-host volatiles in reducing pheromone attraction in European spruce bark beetle *Ips typographus*. Oikos.

[CR209] Zhang Q-H, Schlyter F (2004). Olfactory recognition and behavioural avoidance of angiosperm nonhost volatiles by conifer-inhabiting bark beetles. Agr For Entomol.

[CR210] Zhao T, Krokene P, Björklund N, Långström B, Solheim H, Christiansen E, Borg-Karlson AK (2010). The influence of *Ceratocystis polonica* inoculation and methyl jasmonate application on terpene chemistry of Norway spruce, *Picea abies*. Phytochem.

[CR211] Zhao T, Borg-Karlson AK, Erbilgin N, Krokene P (2011). Host resistance elicited by methyl jasmonate reduces emission of aggregation pheromones by the spruce bark beetle, *Ips typographus*. Oecologia.

[CR212] Zhao T, Krokene P, Hu J (2011). Induced terpene accumulation in Norway spruce inhibits bark beetle colonization in a dose-dependent manner. PLoS One.

[CR213] Zhao T, Axelsson K, Krokene P, Borg-Karlson AK (2015). Fungal symbionts of the spruce bark beetle synthesize the beetle aggregation pheromone 2-methyl-3-buten-2-ol. J Chem Ecol.

[CR214] Zhao T, Ganji S, Schiebe C (2019). Convergent evolution of semiochemicals across kingdoms: bark beetles and their fungal symbionts. ISME J.

[CR215] Zhao T, Kandasamy D, Krokene P, Chen J, Gershenzon J, Hammerbacher A (2019). Fungal associates of the tree-killing bark beetle, *Ips typographus*, vary in virulence, ability to degrade conifer phenolics and influence bark beetle tunneling behavior. Fungal Ecol.

[CR216] Zinecker E (1957) Der große Fichtenborkenkäfer *Ips typographus* L. in seiner Abhängigkeit vom Standort - Site dependendy of the European spruce bark beetle *Ips typographus* Anz Schädlingsk 30: 99–104

